# *Enterococcus* spp.: Is It a Bad Choice for a Good Use—A Conundrum to Solve?

**DOI:** 10.3390/microorganisms9112222

**Published:** 2021-10-26

**Authors:** Mounir Ferchichi, Khaled Sebei, Amine Mohamed Boukerb, Najoua Karray-Bouraoui, Sylvie Chevalier, Marc G. J. Feuilloley, Nathalie Connil, Mohamed Zommiti

**Affiliations:** 1 Unité de Protéomique Fonctionnelle et Potentiel Nutraceutique de la Biodiversité de Tunisie, Institut Supérieur des Sciences Biologiques Appliquées de Tunis, Université de Tunis El Manar, Tunis 1006, Tunisia; mounir.ferchichi@yahoo.fr (M.F.); khaled.sebei@issbat.utm.tn (K.S.); 2 Laboratoire de Microbiologie, Signaux et Microenvironnement (LMSM) EA 4312, Université de Rouen Normandie, 27000 Evreux, France; amine.boukerb@univ-rouen.fr (A.M.B.); sylvie.chevalier@univ-rouen.fr (S.C.); marc.feuilloley@univ-rouen.fr (M.G.J.F.); nathalie.connil@univ-rouen.fr (N.C.); 3 Laboratoire de Productivité Végétale et Contraintes Abiotiques, LR18ES04, Faculté des Sciences de Tunis, Université Tunis El Manar, Tunis 2092, Tunisia; najouakarraybouraoui@yahoo.fr

**Keywords:** lactic acid bacteria, probiotics, enterococcus, virulence, legislation, safety

## Abstract

Since antiquity, the ubiquitous lactic acid bacteria (LAB) Enterococci, which are just as predominant in both human and animal intestinal commensal flora, have been used (and still are) as probiotics in food and feed production. Their qualities encounter several hurdles, particularly in terms of the array of virulence determinants, reflecting a notorious reputation that nearly prevents their use as probiotics. Additionally, representatives of the *Enterococcus* spp. genus showed intrinsic resistance to several antimicrobial agents, and flexibility to acquire resistance determinants encoded on a broad array of conjugative plasmids, transposons, and bacteriophages. The presence of such pathogenic aspects among some species represents a critical barrier compromising their use as probiotics in food. Thus, the genus neither has Generally Recognized as Safe (GRAS) status nor has it been included in the Qualified Presumption of Safety (QPS) list implying drastic legislation towards these microorganisms. To date, the knowledge of the virulence factors and the genetic structure of foodborne enterococcal strains is rather limited. Although enterococcal infections originating from food have never been reported, the consumption of food carrying virulence enterococci seems to be a risky path of transfer, and hence, it renders them poor choices as probiotics. Auspiciously, enterococcal virulence factors seem to be strain specific suggesting that clinical isolates carry much more determinants that food isolates. The latter remain widely susceptible to clinically relevant antibiotics and subsequently, have a lower potential for pathogenicity. In terms of the ideal enterococcal candidate, selected strains deemed for use in foods should not possess any virulence genes and should be susceptible to clinically relevant antibiotics. Overall, implementation of an appropriate risk/benefit analysis, in addition to the case-by-case assessment, the establishment of a strain’s innocuity, and consideration for relevant guidelines, legislation, and regulatory aspects surrounding functional food development seem to be the crucial elements for industries, health-staff and consumers to accept enterococci, like other LAB, as important candidates for useful and beneficial applications in food industry and food biotechnology. The present review aims at shedding light on the world of hurdles and limitations that hampers the *Enterococcus* spp. genus and its representatives from being used or proposed for use as probiotics. The future of enterococci use as probiotics and legislation in this field are also discussed.

## 1. Introduction

Throughout history, the definition of probiotics has undergone constant changes. In 2013, the expert consultation of International Scientists of the International Scientific Association for Probiotics and Prebiotics meeting provided minor corrections and reworded the earlier official definition of the FAO/WHO [1] as “live microorganisms that, when administered in adequate amounts, confer a health benefit on the host” which is now widely accepted and used [2]. Recently, new definitions were added to the probiotic terminology such as parabiotics and postbiotics. The divergence in terms of the definition of probiotics led to an urgent new approach and conceptualization in probiotic terminology to be developed for global usage in the scientific literature [3]. In the last few decades, numerous studies have intensively used probiotic bacterial species in research and scientific investigations [4,5,6,7,8,9,10] revealing a wide range of benefits following probiotics administration, ranging from direct inhibition of sturdy pathogens to improvements of host immune system functions [11,12,13,14,15]. Effective probiotics are typically resistant to bile salts, gastric enzymes, and low pH, which are all traits of enterococci, and do not cause mucosal inflammation or infection. Of note, diverse genera and species of probiotic LAB and non-LAB are used. For instance, *Lactobacillus* and *Bifidobacterium* are the main probiotic genera used by humans, additionally to *Escherichia coli*, *Streptococcus thermophilus*, *Propionibacterium freudenreichii*, *Enterococcus faecalis* and *Enterococcus faecium* and certain species of yeasts such as *Saccharomyces boulardii*. Among these, enterococci have long been used as probiotics and in food fermentation.

Members of the genus *Enterococcus* come third after lactobacilli and streptococcus in the list of LAB largest groups [16]. To date, 58 species and 2 subspecies have been identified within the genus *Enterococcus* [17]. Enterococci are known as the natural inhabitants of the human gastrointestinal tract (GIT) and warm-blooded animals. Such microorganisms have been isolated from plants, water, and soil, probably as a consequence of being in contact with fecal sources and some food products [18]. Hence, bacterial strains belonging to this genus constitute an outstanding part of diverse ecological niches including environmental (soil, water, sewage, plants), clinical and food microbiology [19]. *E. faecalis* and *E. faecium* correspond to the main representatives of this genus [20]. With this versatility, such microorganisms showed tolerance to high salinity levels, even to 6.5% NaCl, to bile salts (up to 40%), to acidic and/or basic conditions (pH up to 9.6) and can survive for 30 min at 60 °C [21]. Different enterococcal species can be employed as biotechnological tools as they can be used as nonstarter LAB flora that helps in ripening and developing aroma properties in cheese production [20,21]. Additionally, some enterococci showed potential probiotic properties and health-promoting capabilities [22,23]. They are also able to produce a wide range of antimicrobial compounds encompassing organic acids (lactic acid, acetic acid), hydrogen peroxide, and bacteriocins so called ‘enterocins’ (bioactive peptides), which limit contamination with pathogens and extend the shelf life of products [24]. In the field of the dairy industry and cheese production, enterococci seem to exhibit a great technological role, especially in the development of organoleptic properties of fermented dairy products when applied as starter or adjunct cultures; this is largely due to their specific biochemical properties such as lipolytic, proteolytic, and esterolytic activities and the ability to ferment citrate to produce diacetyl and several volatile compounds that are sought for their organoleptic properties during the ripening periods [25].

Numerous studies were performed to appraise the probiotic characteristics and potential of enterococci. Diverse strains were found to fit the probiotic prerequisites in terms of acid and bile tolerance, adhesive potential, absence of cytotoxicity, and production of enterocins [5,26]. Likewise, at a commercial scale, several enterococcal strains have been commercialized as probiotics, among which is the *E. faecium* strain 11181 that has been authorized by the European Food Safety Authority (EFSA) Panel as a feed supplement for fattening and enhancing the growth performance of many animals [27], *E. faecium* SF68^®^ (NCIMB 10415; Cerbios-Pharma SA, Barbengo, Switzerland) which is extensively used as a feed supplement for numerous animals and as a pharmaceutical in humans [28], and *E. faecalis* Symbioflor 1 (SymbioPharm, Herborn, Germany) which is also used to prevent or treat diarrhea in pigs, poultry, livestock and pets and to treat recurring illness in the upper respiratory system in humans [16,25].

According to Morandi et al. [29], enterococci are not endowed with ‘GRAS’ status due to the opportunistic and pathogenic nature of some of its members, and also carry some virulence factors, which are present mostly in *E. faecalis* and to a lesser extent in *E. faecium*. Additionally, the EFSA determined that enterococci did not meet ‘QPS’ status [30], a decision that doubted the suitability of these commensals as probiotics. Enterococci are not considered as highly virulent, as they necessitate an immunocompromised host to induce infection, reflecting their opportunistic aspect and how they contribute to cause infections such as bacteremia, intra-abdominal, urinary tract, and nosocomial infections, community-acquired endocarditis and express multi-drug resistance and virulence traits [31,32,33].

Enterococci carrying virulence and antibiotic genes have raised serious worries about their use as probiotics [34]. Defined as effector molecules, the virulence factors show high potential in terms of enhancement of the disease-causing effect of bacteria via adhesion to the host tissue, colonization, increasing bacterial migration and host immune regulation [35]. A whole list of enterococcal virulence determinants has been identified and reported encompassing aggregation substance, cytolysin, gelatinase, hyaluronidase, enterococcal antigen A, surface adhesins, and sex pheromones [36,37]. In particular, the spread of vancomycin-resistant enterococci (VRE) significantly increased mortality and disease burden due to enterococcal infection [38], raising safety concerns for the use of enterococci as probiotics [25]. However, numerous representatives are used as probiotics and in production of feed additives in order to prevent diarrhea and/or to enhance growth in livestock [39]. Such a situation has created a prerequisite for new drastic legislation of probiotics in terms of safety for the purpose of discerning between safe and potentially pathogenic strains. Herein, we sum up the main risks associated with enterococci as probiotics and underscore the dual and controversial traits between opportunistic pathogens and promising probiotics, with emphasis on the safety regulation of this genus.

## 2. Enterococci in Food—Enterococci as Probiotics

Health benefits that confer probiotic microorganisms include modulating immunity, enhancing intestinal barrier function, or altering pain perception [40]. Generally, the big portion of the probiotic kingdom of intestinal origins belongs to the LAB group especially to genera of *Bifidobacterium* and *Lactobacillus*, whereas enterococcal strains are scarcely used [39]. In this framework, numerous investigations have been performed to assess the probiotic traits of Enterococcal strains and their beneficial animal and human health-promoting effects [5,7,10,26,41,42]. Therefore, enterococcal species have been used as probiotics for a wide range of purposes and applications encompassing the pharmaceutical industry, human and veterinary medicines, and the food industry since some probiotic enterococci can be used in the production of functional foods [40]. Table 1 recaps the most relevant enterococcal preparations used as probiotics for human consumption.

It is pertinent to note that due to the lack of safety data and legislation, only a limited number of enterococci are commercialized but not yet labelled with ‘GRAS’ status [39]. For instance, different probiotic formulas, containing *E. faecium* M74 and *E. faecium* SF-68, have been reported to be effective and safe. Amongst these preparations, Cernivet^®^ and FortiFlora^®^ (containing *E. faecium* SF68^®^, Cerbios-Pharma SA, Barbengo, Switzerland), and Symbioflor^®^ 1 with *E. faecalis* (Symbiopharm, Herborn, Germany) are the most relevant and commercialized [45,46]. Moreover, numerous enterococcal strains have been described as useful for many health features or technological applications in food systems or in human and/or animal health, including *E. durans* M4-5 (production of butyrate as short chain fatty acids (SCFAs), induction of significant anti-inflammatory effects and contribution to the intestinal epithelium integrity), *E. durans* LAB18s (source of dietary selenium supplementation), *E. durans* KLDS 6.0930 and *E. faecium* M74^®^ (reduction in serum cholesterol levels), *E. mundtii* ST4SA (production of bacteriocins), *E. faecium* LCW 44 and *E. durans* 6HL (production of antimicrobial compounds against Gram-positive and Gram-negative bacteria) [25].

Regarding feed regulation, the EFSA authorized certain strains of enterococci for use as silage additives and dietary supplements. For instance, some enterococcal probiotics were included in the group of gut ecosystem enhancers, antibiotic replacers, and feed additives for stabilizing the microbial communities of the digestive tract in both monogastric and ruminant animals [14]. A wide range of enterococci strains and the bioactive peptides produced, also known as enterocins, have been reported and approved as promising probiotics in the husbandry industry in terms of boosting growth performances, improving health, fighting sturdy pathogens, enhancing metabolic efficiency and immunological parameters, alleviating antibiotic-induced diarrhea, and maintaining animal integrity. Such beneficial effects are addressed to almost all types of animals ranging from farm animals to aquaculture and even in pets. In the latter field, Enterococci have been used as beneficial microbes for dogs on the basis of their tolerance to bile, adhesion activity, microbial antagonism and their impact on serum cholesterol and alanine aminotransferase [47]. Accordingly, diverse probiotic formulas, containing enterococcal strains in solo or combined with other species, have been developed and commercialized. Table 2 sums up enterococci probiotic formulas in the nutrition of livestock.

Despite several safety issues, and the non-obtention of GRAS status by enterococcal strains, numerous representatives of this genus are well characterized and have served as starter cultures, co-cultures, or protective cultures in food industry and/or probiotics due to their positive traits. *Enterococcus* spp., a two-sided coin, functioning concomitantly as a good candidate probiotic and an opportunistic pathogen, represents a critical topic in need of continuous debate focusing on the question of whether enterococci are safe for probiotic use, a question that still remains difficult to answer.

In terms of probiotic attributes, *Enterococcus* spp. encounters numerous obstacles to fulfill these criteria; in particular, in terms of pathogenicity, the horizontal transfer of virulence determinants, and the constantly increasing number of enterococcal infections in recent decades are escalating the concerns [31,40]. A surprising criterion of this genus is that it has never been suggested as a foodborne pathogen [48]. Relatedly, after being suspected as a causative agent of foodborne illness in 1926, a wide range of investigations on enterococci, in particular *E. faecalis* and *E. faecium*, encompassing experiments and trials on animals and human volunteers were performed to confirm that enterococci cause foodborne illness; however, studies yielded negative results because these bacteria are generally identified together with other pathogens such as staphylococci [49]. Subsequently, enterococci have emerged as nosocomial- and community-acquired pathogens rather than foodborne pathogens [50]. Accordingly, as a critical conundrum, the safety of enterococci must be meticulously assessed on a case-by-case basis. Effectively, when selecting an enterococcal potential probiotic strain, various aspects should be considered including the safety aspect, techno-functional properties, and beneficial features. Since the probiotic effect is strain dependent, it should be drastically characterized (phenotypically and genotypically) and must be safe and free of any pathogenicity such as the absence of virulence determinants and acquired antibiotic resistance genes [1,51]. For the latter, a whole list has been created of desirable characteristics for probiotic strains encompassing the ability to survive and retain viability in harsh gastrointestinal tract conditions of a healthy human (low acidity, pepsin presence, pancreatin, bile salts), their inability to translocate the intestinal mucosa, their susceptibility to phagocytic killing, and the potential to produce antimicrobial substances such as enterocins. A further considerable aspect for potential enterococcal probiotics is that they should have a limited ability to exchange DNA in vivo [40].

## 3. Virulence Determinants Associated with Enterococci

### 3.1. Colonization-Related Virulence Factors

Enterococci, as opportunistic pathogens, are endowed with a whole arsenal of virulence factors (Table 3). According to Tomita and Ike [52], enterococcal strains are endowed with strong adhesive abilities allowing high adhesion to the host’s tissues. This criterion, coupled to their resistance to low acidity and high concentrations of bile salts [53], makes enterococci amongst the most common types of bacteria colonizing the colon. These proteins, also known as adhesins, permit these bacteria to bind to mucosal membrane receptors or to the extracellular matrix proteins, facilitating colonization of the epithelium [34]. In case of not binding, they would be removed by intestinal peristaltism. It has been noted that the phenomenon of colonization in itself has never been a proof of pathogenicity but coupled with other factors of virulence and with the presence of a number of resistance genes, they may become potentially harmful. Virulence determinants involved in promoting colonization include aggregation substance (AS), collagen-binding protein (Ace), cell wall adhesin (Efa A), and enterococcal surface protein (Esp) [54,55].

As the most abundant representatives on the *Enterococcus* spp. genus, *E. faecalis* and *E. faecium* are invasive because of their capacity to translocate intact mucosal barriers and access host tissues. Accordingly, *E. faecalis*, in solo, persists in phagocytic cells and produces extra-cellular superoxide [56,57]. This latter phenotype has been tightly associated with genotoxicity, chromosomal instability, and neoplastic transformation [58,59,60]. In terms of aggregation, the wide spectrum of virulence determinants allows enterococci to adhere to host cells, to invade mucosal barriers, to resist phagocytosis, and to regulate host immunity leading to cellular destruction.

It is relevant to know that accurate genome sequencing and analysis of a wide range of potential probiotic and/or probiotic enterococci unveiled the presence of several virulence factors. For instance, Domann et al. [61] revealed the presence of several virulence determinants after the genome annotation of *E. faecalis* Symbioflor 1 probiotic strain. Genes encoding for Cyl, Esp, and GelE were absent, although other virulence factors were present, among which were AS, collagen adhesion protein, and oxidative stress resistance. Likewise, in 2015, Natarajan and Parani [44] conducted a genome sequencing study of the probiotic *E. faecium* strain T-110, in which they exhibited the presence of few enterococcal virulence factors. According to Arias and Murray [31], many of them have been well characterized since the early 1990s. Because of the importance of virulence when considering enterococci as potential probiotics, several of the more thoroughly characterized enterococcal virulence traits are described below. Regarding this subject, excellent reviews have been written providing additional details about *Enterococcus* spp., its representatives, the most characterized enterocins and the associated virulence determinants [21,25,31,43,62,63,64,65].

#### 3.1.1. Aggregation Substance

The AS represents the first enterococcal surface protein to be described. As it often acts as a virulence factor and it transfers antibiotic resistance genes, it is still a subject of current intensive investigations. AS is a 137 kDa surface protein encoded by *prg*B on pheromone-responsive plasmids and endowed with a hairpin-like structure [66,67,68]. In 2005, Dramsi and coworkers [69] showed that the strongly conserved motif LPXTG corresponds to a pivotal part of the aggregation substance’s molecule and its distinctive sequence is regarded as the site of recognition and cleavage by sortases which bind via a covalent bond to the cell wall. The AS comprises a range of highly homologous adhesins, encoded on large conjugative plasmids transferred in a so-called facilitated conjugation system, mediated by sex pheromones [54]. This protein is involved in several physiological events. The adhesin domain of AS is crucial for cellular aggregation prior to plasmid exchange, conjugative DNA transfer, biofilm formation and virulence [70]. Two pilot studies performed by Schlievert et al. [71] and Chuang et al. [72] on animal models suffering from endocarditis unveiled the presence of AS-positive *E. faecalis* strains favoring increased size and mortality compared to AS-negative strains. Regarding sex pheromones, these latter are short, hydrophobic peptides, which enter the AS and interact with a specific conjugative plasmid [73]. The process is of particular significance in the conjugative transfer of genes between cells. In the presence of pheromones secreted by the recipient’s cells, the donor’s cells synthesize the AS which binds to a related enterococcal binding substance (EBS) ligand on the recipient cell surface contributing to lethality in experimental models [71,74]. Such a process results in the formation of large conjugative aggregates consisting of bacterial cells, which enables the exchange of genetic material between cells. Surprisingly, the AS protein showed traits of a superantigen in the presence of a specific ligand with a structure akin to that of teichoic acid [75,76]. Additionally, such a molecule may ensure a role in propagation within a variety of plasmids, on which other specific determinants of enterococci virulence are encoded, such as cytolysin and genes of antibiotic resistance. To sum up, the AS and cytolysin can act synergistically, hence boosting the notorious reputation of strains in terms of virulence via switching on cytolysin regulation in the quorum-sensing system, making it possible to cause irreversible damage in deeper tissues [53,77].

About 20 pheromone-dependent plasmids were found in enterococci, encoding antibiotic resistance genes together with AS genes. In 2007, studies conducted by Clewell [78] and Dunny [79] discovered the conjugative plasmids associated with genes responsible for the production of AS proteins: pAD1 (Asa1 protein), pPD1 (Asp1 protein) and *pCF10* (Asc 10 protein). Hendrickx and collaborators [80] demonstrated that the genes responsible for production of AS proteins are strongly conserved showing about 90% similarity. However, the *asa*373 gene that is located on the pAM373 plasmid displays a sequence that is considerably different from those mentioned above. Moreover, it has been disclosed that only Asa337 is capable of binding to the recipient’s cells that lack the active binding substance [81]

#### 3.1.2. Endocarditis Specific Antigen—EfaA

EfaA (endocardis antigen) corresponds to a protein with a molecular weight of about 34 kDa. This polypeptide is encoded by the *efa*A_fs_ gene in *E. faecalis*, and by *efa*A_fm_ in *E. faecium* [82,83]. According to Abrantes et al. [84], the *efa*A gene is part of the *efa*CBA operon which encodes an ABC transporter (permease), regulated by magnesium ions. It is pertinent to note that the EfaA protein has been reported as homologous to the adhesins of the cell wall of streptococci [85]. Accordingly, genetic approaches were able to prove that homologous genes to *efa*A are present in strains of *E. avium*, *E. asini*, *E. durans* and *E. solitarius* [86,87].

#### 3.1.3. Surface Protein—Esp

The largest identified enterococcal protein, also known as ‘Esp’ for Enterococcal surface protein, has a molecular weight of 202 kDa and is encoded by the *esp* gene which is located on the pathogenicity island (PAI), which itself contains genes coding for proteins responsible for the active outflow of antibiotics [88,89]. This location may be the result of a horizontal gene transfer between *E. faecalis* and *E. faecium*. Esp is more frequent among pathogenic isolates of *E. faecalis* than commensal strains. This protein contributes to colonization and persistence in urinary tract infection suggesting a potential role in virulence [90,91]. Likewise, Esp, which is highly expressed in *E. faecium*, is regulated by the *ebr*B gene, and contributes to biofilm formation and intestinal colonization in mouse models [92]. Additionally, it promotes colonization of *E. faecium* on heart valves and contributes to virulence in endocarditis [93].

Diverse studies have demonstrated the presence of structural similarities between Esp and other proteins of Gram-positive bacteria that are associated with biofilm formation such as the C-α in β-hemolytic protein in *Streptococcus agalactiae* encoded by the bca gene, R28 in *Streptococcus pyogenes* and Bap in *Staphylococcus aureus* [80,94,95]. In this context, several inquiries about the contribution of Esp in biofilm formation have been confirmed. Nevertheless, despite the enhancement of biofilm formation by Esp, this surface protein is not pivotal to the phenotype. For instance, *E. faecalis* strain OG1RF is Esp-negative yet it still produces biofilm [32,96]. Biofilms on implanted prosthetic devices (e.g., artificial joints, heart valves, and intravascular catheters) render these infections nearly impossible to cure revealing their critical role in the exchange of genetic material between cells and the increase in their resistance to antibiotics [53,95,97]. In 2008, Billstrom and collaborators [98] revealed that the occurrence of the *esp* gene in *E. faecium* is correlated with resistance to ampicillin, ciprofloxacin, and imipenem. Likewise, a significant relation between Esp and resistance to vancomycin has been suggested. For instance, Ochoa et al. [99] noted that 83.3% of the vancomycin-resistant clinical strains of *E. faecium* possessed the *esp* gene, reflecting its possible contribution in multidrug resistance [98]. In 2004, Oancea et al. [100] showed that *esp* can be transferred between strains of *E. faecium* via plasmid conjugation, and also between strains of *E. faecalis* by chromosome–chromosome transposition.

### 3.2. Virulence Factors Affecting Host Tissues

According to Goguen et al. [101], bacterial proteases can contribute to pathogenesis through altering host tissues or activating virulence factors. For instance, it has been shown that after the colonization process, pathogenic strains of *Enterococcus* spp. secrete toxic substances with a destructive effect on the host’s tissues. Virulence determinants secreted by enterococci encompass: cytolysin (Cyl), gelatinase (GelE) and hyaluronidase (Hyl).

#### 3.2.1. Gelatinase—GelE

Gelatinase is defined as an extracellular, zinc-dependent metallo-endopeptidase, with a molecular weight of about 30 kDa. This enzyme has shown a high potential to hydrolyze mainly gelatine, elastin, collagen, hemoglobin, as well as other bioactive peptides such as proteins bound to pheromones [85]. Diverse mechanisms have been identified in order to clarify and unveil the pathogenic effects of GelE. Park and collaborators [102] reported the potential of this protease to enhance bacterial invasion via cleaving human complement C3 and inhibiting phagocytosis by polymorphonuclear leukocytes. Additionally, GelE can degrade polymerized fibrin as another potential mechanism for virulence. It also may eliminate misfolded bacterial surface proteins and extracellular pheromones leading to an interaction with protease-activated receptor 2 (PAR2) to induce intestinal permeability [103]. Disruption of GelE leads to increased chain length of enterococci to 5-10 cells, reflecting a potential role in enterococcal cell division and morphology [104]. In 2004, two studies were performed by Hancock and Perego [105] and Pillai et al. [106] in which gelatinase and its regulation were deeply investigated. This enzyme is encoded by the chromosomal *gel*E gene for which expression is regulated by the transmembrane protein FsrB. This gene is part of the *fsr*ABC cluster, encoding the regulatory protein FsrA, FsrB—pheromone transporter GBAP and histidine kinase FsrC. Deletions within the locus *fsr* render these bacteria unable to produce gelatinase leading to decreasing virulence [107]. In addition, such mutants were also affected in biofilm formation reflecting a significant reduction in the biofilm synthesis by 28–32% [108]. The *gel*E gene is regulated by the quorum-sensing system which relies on the gelatinase biosynthesis activation pheromone (GBAB) [109,110]. This gene is present in both clinical and food-derived enterococcal strains. It usually occurs in *E. faecalis* and in individual strains of *E. faecium* [82]. To summarize, it is relevant to note that this protease also contributes to biofilm formation [105].

#### 3.2.2. Hyaluronidase—Hyl

Hyaluronidase is a protein that is mostly found in *E. faecium*, with an approximative molecular weight of 45 kDa and encoded by the chromosomal *hyl* gene [85]. The *hyl* gene has been usually identified in *E. faecium* and it occurs regularly in *E. faecalis* [111]. It has also been found in other strains isolated from food encompassing *E. casseliflavus*, *E. durans* and *E. mundtii* [112]. Hyl is homologous with hyaluronidases of other cocci such as *S. pyogenes*, *S. pneumoniae* and *S. aureus*. Such proteolytic protein is implicated in the degradation of mucopolysaccharides that connect tissue and cartilage and, therefore, in spreading bacteria.

It has been widely believed that bacterial strains of clinical origin usually contain more virulence determinants than those isolated from other sources, including foods and food products. The latter have been reported as an indirect source of infection with a significant implication in promoting the spread of virulence genes [82,84,113]. According to the data mentioned above, Huddleston [114] reported that Enterococcal strains are capable of exchanging genetic material via conjugation and the process frequently occurs in the GIT. For instance, diverse enterococcal virulence determinants, including hemolysin-cytolysin, adhesive substances or antibiotic resistance, can be transferred by the mechanism of gene exchange, e.g., the case that one plasmid may contain genes encoding pheromones, antibiotic resistance and other virulence factors [115,116]. Accordingly, it seems reasonable to monitor the presence of virulence factors in strains of the *Enterococcus* spp. genus isolated from food.

## 4. Other Enterococcal Virulence Factors

### 4.1. Sex Pheromones

In 2002, Clewell and coworkers [117] demonstrated that enterococcal strains possess a mechanism of plasmid accumulation based on the production of chromosomally encoded genes of the sex pheromones encoded by—*cpd*, *cob*, *ccf*, *cad*. These are defined as small peptides, composed of 7 to 8 amino acids, which mediate the conjugative transfer of plasmids between cells [118]. It is relevant to know that pheromones secreted by recipients are donor-specific and induce the expression of conjugative operons of its plasmid. Usually, an enterococcal strain is not limited to the secretion of one type of pheromone. It may secrete several different pheromones. Apart from pheromones themselves, every single pheromone-dependent plasmid encodes for the secretion of peptides which act as competitive inhibitors of the corresponding pheromone. Two studies carried out by Clewell [119] and Dunny et al. [74] demonstrated that the binding of pheromones to receptors on the surface of the donor’s cells permits the transduction of this signal and induces the gene of the AS. Expression of the *asa1* gene results in the formation of cell aggregates, which enables effective plasmid transfer [117]. Additionally, amongst the different roles of pheromones, Bhardwaj and his collaborators [120] revealed that these peptides can be chemically attractive to human neutrophils, they may initiate inflammatory conditions and they even induce the production of superoxides. These latter, as a specific toxic compound produced by *E. faecalis*, have been reported for the first time by Falcioni et al. [121]; such compounds have never been detected in *E. faecium* strains before. These substances can also behave as a mutagen [122]. In the same framework, Wang and Huycke [58] demonstrated that *E. faecalis* caused chromosomal instability in eukaryotic cells via superoxide production, while using a single-nucleotide polymorphism array. In the field of cancerology, DNA instability is widespread within different types of solid tumors, including colorectal cancer (CRC), and directly related to poor prognosis and metastasis [123,124]. For instance, *E. faecalis*-induced tetraploidy may be similarly linked to increased chromosomal instability [65].

### 4.2. Biogenic Amines

In addition to the aforementioned list of virulence factors, biogenic amines (BAs) seem to play a critical role in the pathogenesis of enterococci. Defined as organic basic compounds, these substances may occur in many foods such as vegetables, fruits, beer, wine, cheese, eggs, fermented sausages and fish products. According to Giraffa [116] and Kucerova et al. [125], the most relevant BAs found in foods and food products are tyramine, histamine, putrescine, tryptamine, p-phenylethylamine, spermine, spermidine and cadaverine, where they may display a high risk of food intoxication. Such foodborne disease may be of critical clinical concern and are characterized by symptoms of increased blood pressure, vomiting, severe headaches and allergic reactions. Of note, fermented foods correspond to the main reservoir of BAs due to the action of amino acid decarboxylases of microbial agents included as starter cultures or present as part of contaminating microflora. For instance, in 2017, Aspri et al. [126] reported the ability of enterococci to form BAs during growth in dairy products, particularly in the case of tyramine which is often considered to be the only biogenic amine formed by enterococci. Nevertheless, this result has been rebutted by the findings of other investigations which demonstrated the ability of enterococci to produce other BAs, via histidine and tyrosine decarboxylase activities, during their growth in milk and other dairy products [125,127]. However, the production of BAs is dependent on the magnitude of enterococcal growth and enterococci rarely reach sufficient cell numbers in cheese and milk to be considered a threat regarding the production of BAs [128]. Although the production of BAs by enterococci has been reported, these compounds have yet to be implicated in enterococcal disease. Thus, although an important consideration, it would seem judicious to assess these capacities in enterococci isolated from foods and also in those used as starter cultures of probiotics.

## 5. Are There Any Other Limitations to the Use of Enterococci as Probiotics?

In addition to the above-mentioned virulence determinants, enterococci are endowed with a notorious reputation in terms of sturdy phenotypes that render them inadequate as probiotics and for probiotic use. Amongst these phenotypes are resistance to innate immunity, an innate ability to translocate intact mucosal barriers, high persistence in phagocytic cells, facile exchange of genetic elements, acquired resistance to many antibiotics, and an association with CRC. In the next sections, we develop the discussion further by taking *E. faecalis* as an example.

### 5.1. Resistance to Innate Immunity

Escape to innate immune cells is pivotal for pathogens responsible for invasive infection. In this context, Asahara et al. [129] showed that a wide range of probiotics, including lactobacilli, are readily killed by phagocytosis following translocation. However, *E. faecalis* demonstrates a remarkable resistance to innate immunity with a high persistence in macrophages. Two pilot investigations led by Wells et al. [130] and Zou and Shankar, [131] showed that in a murine peritoneal infection model, antigen-presenting cells were unable to efficiently eliminate *E. faecalis*. These cells are able to survive in vitro for more than 3 days while a proportion exceeding 90% of *E. coli* and *Lactococcus lactis* strains are killed within 24 h [131]. In terms of resistance to oxidative killing, *E. faecalis* possess various genetic determinants implicated in this phenomenon. Of note, *E. faecalis* tolerance to hydrogen peroxide (H_2_O_2_) contributes to the improvement of their survival within murine peritoneal macrophages. Numerous studies have succeeded in revealing that such tolerance is controlled by proteins coded by *hyp*R, *per*R, and Ara-C type transcriptional regulators [132,133,134]. These gene products likely control expression of diverse antioxidant genes that include NADH peroxidase, alkyl hydroperoxide reductase, and thiol peroxidase [135].

Regarding the production of extracellular capsules by enterococci, particularly *E. faecalis* strains, these bacteria are endowed with this feature, and their extracellular capsules are classified into 21 unique serogroups [136]. It is significant to note that capsular antigens are sugar polymers that render strains resistant to phagocytosis and boost their persistence in a mouse model of subcutaneous infection [137]. In 2009, Thurlow et al. [138] identified an operon for capsular synthesis in a wide range of clinical strains of *E. faecalis* implying the potential role of these capsules in infection. Despite the scarcity of data about foodborne *E. faecium* capsules, it has been shown that clinical isolates that are resistant to phagocytosis presumably produce capsules [139]. Recently, Zou and Shankar [131] proved that *E. faecalis* is resistant to phagosome acidification and does not induce autophagy.

### 5.2. Intestinal Translocation

According to Liong [140], intestinal translocation represents a critical challenge to the safety of any probiotic enterococcal strain. Within this field, a wide range of studies have demonstrated the relevant contribution of bacteria, particularly *E. faecalis* and *E. faecium*, to the translocation of mucosal surfaces in order to colonize and/or cause infection in regional lymph nodes, liver, and spleen [141,142,143]. For instance, in 2008, Allen and collaborators [144] showed, via a short-term colonic ligation model, that *E. faecalis* are able to activate NF-κB signal transduction in colon macrophages. Such an effect was noticed using heme-starved bacteria, a growth state that stimulates extracellular superoxide production. This action at a distance, i.e., luminal bacteria activating mucosal immune cells, requires epithelial translocation through specialized intestinal cells termed M (microfold) cells or by the luminal sampling of bacteria through transepithelial dendrites extending from colon macrophages [145,146]. Of note, intestinal translocation of enterococcal strains is firmly correlated with numerous ailments and disorders. In fact, translocation of *E. gallinarum* to the liver stimulates auto-immune responses in a systemic lupus erythematosus (SLE)-like mouse model and patients with SLE and auto-immune hepatitis. Treatment with antibiotics and by vaccination has permitted the prevention of *E. gallinarum*-induced auto-immune responses and mortality [143]. However, Knoop et al. [147] showed that antibiotics have the potential to promote the translocation of *E. faecalis* and induce inflammation. In a pilot investigation performed on a murine model of ischemic stroke, the translocation of *E. faecalis* into tissues encompassing lung, liver, spleen, and mesenteric lymph nodes was highly linked with post-stroke infection [148].

According to Wang and Huycke [58], the translocation of *E. faecalis* may also be firmly associated with colorectal carcinogenesis. In fact, this team revealed that in vitro activation of colon macrophages by *E. faecalis* led to the production of clastogens (or chromosome breaking factors) that, in turn, caused chromosomal instability in neighboring cells through a bystander effect. This sequence results in epithelial DNA damage characterized by aneuploidy, tetraploidy, G2M cell cycle arrest, and cellular transformation [59,149]. Two diffusible factors mediate the bystander effect: 4-hydroxy-2-nonenal (4-HNE) and tumor necrosis factor alpha (TNFα). In the same context, in the year 2012, two pilot studies were conducted by Wang et al. [149] and Yang et al. [150] in which they succeeded in elucidating the role of 4-HNE as a mutagen and clastogens, and the role of TNFα as a promotor of colon epithelial cell proliferation. It has been shown that superoxide and cyclo-oxygenase-2 may ensure a contribution to the bystander effect as superoxide dismutase and cyclo-oxygenase inhibitors confer protection [58,60,151]. To sum up, these findings highlight several worrying predispositions of translocating and mutagenic enterococcal strains, providing further arguments against consideration of *E. faecalis* as a probiotic.

In terms of CRC, numerous works have validated the association of *E. faecalis* with murine colitis and this type of cancer via the use of interleukin-10 (IL-10)-deficient mice [60,152,153]. Of note, data about the correlation between *E. faecalis* and human CRC are scarce. Nevertheless, intensive efforts in this field succeeded in linking the presence of *E. faecalis*, as the predominant species, in fecal samples and colon tissues (tumor and adjacent normal tissues) from patients with CRC compared to healthy controls [154]. Likewise, recent investigations on clinical cases of enterococcal bacteremia and endocarditis have suggested a tight link between enterococcal strains and CRC [155,156,157]. In the same way, Pericàs et al. [158] revealed the link between colorectal neoplasms in more than half of patients with *E. faecalis* endocarditis, implying a firm connection between this bacterium and colorectal cancer. Finally, CRC patients colonized with *E. faecalis* have been correlated with specific cancer phenotypes that are characterized by upregulated CpG island methylator phenotypes, micro-satellite instability, and pathways involved in inflammation and DNA damage [159]. Together these findings imply a critical role for *E. faecalis* in CRC.

### 5.3. Transfer of Virulence Determinants and Antibiotic Resistance Genes

According to O’Driscoll and Crank [160], enterococci belong to the group of the most common nosocomial pathogens that could cause a wide range of critical infections and ailments including bacteremia, endocarditis, urinary infections, intra-abdominal and pelvic infections, foodborne diseases and even central nervous system infections. Significantly, nearly 80% of the aforementioned infections were associated with *E. faecalis* [161]. Enterococcal strains, previously viewed as microbes of minimal clinical impact, have emerged now as common opportunistic pathogens of humans [162]. The pathogenicity of these microorganisms is presented via a whole arsenal of virulence determinants and the constant increase in antibiotic-resistant strains, particularly, vancomycin-resistant enterococci (VRE) [115,140,163]. Accordingly, *Enterococcus* spp. represent a critical heavy burden towards public health, especially when identified as the main causative agent of infection and/or disease, specifically in immunocompromised subjects [164]. In 2010, Brilliantova and coworkers [165] demonstrated that infections caused by enterococcal strains originated from the intestinal microbiota of the patient and can be transferred from one subject to another or can be acquired by the consumption of contaminated food and water. This bacterial genus shows an unexpected potential of transferring the antibiotic-resistant genes in order to produce AS, cytolysin and gelatinase that are common enterococcal virulent traits [166].

Regarding the antibiotic resistance of numerous enterococcal strains to conventional antibiotics, this represents another critical virulence feature which strongly boosts the pathogenicity and the virulence of *Enterococcus* spp. via making them sturdy opportunistic microorganisms in nosocomial infections [167,168,169]. In this context, it has been shown that continuous exposure to antibiotics, particularly, their intensive use in human and veterinary medicines in terms of prophylaxis, therapy and/or growth promoters, has led to an imminent increase in the occurrence of multi-drug resistant enterococcal strains. Consequently, this multi antibio-resistance becomes a serious public health issue. Furthermore, it has been revealed that enterococci are intrinsically multi-drug resistant, reflecting a resistance to cephalosporins, sulphonamides, lincosamides, β-lactams, and aminoglycosides, located in the chromosomes [116,170]. Nevertheless, acquired resistances in enterococci from other microorganisms occurred via plasmids or transposons, and they could be observed towards ampicillin, chloramphenicol, erythromycin, fluoroquinolones, penicillin, tetracycline, aminoglycosides (gentamicin, kanamycin, and streptomycin), glycopeptides (especially vancomycin) and even oxazolidinones [45,171,172]. It is pertinent to note that antibiotic resistance does not alter enterococcal virulence per se or directly promote pathogenesis. Though, widespread resistance does promote colonization and superinfection, limits treatment options, and renders cure less likely. Genetic determinants of antibio-resistance are frequently detected in enterococcal strains and are readily found in probiotic strains, food isolates, and certain commensal *E. faecium* strains (clade B) that are usually considered vancomycin susceptible [115,173,174].

Regarding vancomycin resistance, this is of special interest because vancomycin-resistant enterococci (VRE) were known to cause critical infections and ailments that could not be treated with conventional antibiotic therapy [175]. That is why VRE represented (and still represents) a real challenge to clinicians since this antibiotic, vancomycin, has traditionally been considered as the “drug of last resort” in the treatment of enterococcal infections as it is often used to replace ampicillin, penicillin, and aminoglycosides in patients with allergies [176]. Accordingly, an arsenal of new drugs was developed, tested and assessed as potential alternatives to vancomycin encompassing everninomycins, daptomycin, oxazolidinones, and quinupristin-dalfopristin [116]. In the same framework, avoparcin, a glycopeptide similar to vancomycin, was widely used as a growth promoter in livestock feed in the 1970s and is considered as the most famous example of the acquisition and transfer of antibio-resistance in enterococci. During the 1990s, vancomycin-resistant enterococci were detected in foods, food products and emerged as colonizing strains in animals and healthy people. Likewise, numerous investigations conducted in European and American countries reported that VRE colonization arises in the community besides human reservoir; animal, environmental, and food reservoirs could act as community sources for VRE outside the health care setting [116]. More recently, Wegener [177] announced that VRE appeared as the leading nosocomial sturdy pathogens around the globe. In fact, there are six known genes of glycopeptide resistance in enterococci: *van*A, *van*B, *van*C, *van*D, *van*E, and *van*G [177]. In 2018, Leong et al. [178] succeeded in detecting vanA or vanB resistance genes in 222 *E. faecium* clinical isolates. This cautionary tale demonstrated that antibiotic use in animal husbandry can rapidly spread resistant enterococci via the food chain to humans and the clinic. Moreover, vancomycin-resistant enterococci could also occur in human outside hospitals confirming that a transfer of resistance genes between animals and humans or a clonal spread of resistant strains could explain this prevalence. Hence, VRE could reach foods via the path of environmental contamination from diverse sources, wastewater from sewage treatment, livestock feces, and manure from poultry farms [116,179]. In harmony with these findings, a long list of other antibiotic-resistant enterococcal strains has been found amongst food, animals and the environment worldwide. For instance, several studies have detected high gentamicin, kanamycin, streptomycin, tetracycline and glycopeptide resistance within enterococcal strains (*E. faecalis*, *E. faecium*, *E. casselifavus*, and *E. gallinarum*) isolated from bovine mastitis (80%), chickens (62–64%), pigs (57%), food of animal origin (e.g., white and red meats), uncooked food (e.g., lettuce), sewage, and water [174,180,181,182]. It is relevant that the emergence of this high antibiotic resistance in all of the above-mentioned reservoirs and environments implies the inter-strain transmission of genetic resistance determinants in the intestinal tract and through in vitro conjugation models. Such a deduction has been proved via several studies [183,184,185,186,187].

When considering enterococci as probiotics, their inherent potential to exchange resistance genes is a salient feature. In 2015, a study performed by Topcuoglu et al. [188] clinically investigated the presence of VRE in different probiotic products containing *Lacticaseibacillus casei*, *Lacticaseibacillus rhamnosus*, *Lactiplantibacillus plantarum*, *Bifidobacterium lactis*, fructooligosaccharide, galactooligosaccharide, colostrums and lactoferrin in premature infants. It showed that a shocking VRE outbreak incidentally occurred reflecting the critical potential of probiotics to enable antibiotic resistance gene transfer. Accordingly, these findings increase new safety issues for enterococcal probiotics, in particular for patients with immune-compromised health conditions. In the context of inter-strain transmission of resistance genes, Lund and Edlund [185] discovered the potential of antibiotic resistance transfer to probiotic strains of *E. faecium*. Likewise, in 2019, Li and colleagues [189] demonstrated that a vancomycin resistance gene (*van*A) was readily transferred between probiotic *E. faecium* and *E. faecalis* strains during fermentation of a soybean meal. In 2010, Palmer et al. [190] revealed that high-efficiency transfer of resistance to macrolides, tetracyclines, and glycopeptides within *E. faecalis* and *E. faecium* arises via pheromone-responsive plasmids. These plasmids, as highly evolved extra-chromosomal genetic elements, may play a double role concomitantly, encoding diverse resistance determinants and containing virulence genes for Cyl, AS, and the biofilm enhancer in *Enterococcus* (bee) and, as with the PAI, contribute to the spread of virulence factors. Unlike *E. faecalis* and *E. faecium*, *E. gallinarum* and *E. casseliflavus* exhibit intrinsic vancomycin resistance that is chromosomally encoded by the *van*C gene [191].

Relatedly, Palmer and Gilmore [192] succeeded in exhibiting the different pathways used by enterococci to spread and transfer antibiotic resistance genes including broad-host range plasmids, composite, conjugative, and Tn3-like transposons, and even via numerous bacteriophages. They also determined that the acquisition of mobile DNA elements in multi-drug resistant isolates of *E. faecalis* and/or *E. faecium* is highly associated with the absence or deficiency in CRISPR/cas loci. ‘CRISPR’ for Clustered Regularly Interspaced Short Palindromic Repeats and its associated protein (Cas) represent adaptive immune systems which may occupy a defensive role for the genome against horizontal acquisition of foreign DNA. This DNA potentially transfers resistance genes and factors, not just between enterococci but also to non-enterococcal Gram-positive and -negative bacteria, hence expanding the reservoir of these determinants [193]. Recently, a few studies have succeeded in detecting a *Clostridium botulinum* neurotoxin (BoNT)-like toxin gene on a conjugative plasmid in some selected *E. faecium* strains. Such discoveries imply the potential horizontal transfer of a dangerous toxin gene [194]. One of the BoNT-positive strains was phylogenetically similar to the probiotic *E. faecium* T-110 strain [195]. Such findings pose new critical concerns that complicate the situation in terms of using enterococci as probiotics.

## 6. Virulence Potential of Foodborne Enterococcal Strains: Myth vs. Reality

According to Sharifi et al. [196], accurate knowledge and understanding of the virulence determinants and features of circulating enterococcal strains may help to comprehend the complex pathogenic process of these opportunistic microbes. It is important to note that pertinent data on the virulence of enterococcus isolated from foods and food matrices are still scarce. This paucity is due to diverse factors. In fact, scientific investigators have revealed that the virulence of this genus seems to depend on the species rather than on the source of their isolation. Accordingly, *E. faecalis* represents the candidate with a whole arsenal of virulence determinants, more than in isolates of other species. In 2001, Franz and coworkers [115] presented significant levels of *E. faecalis* and *E. faecium* strains isolated from food with at least one virulence factor, 78.7% versus 10.4%, respectively. Likewise, Cariolato et al. [197] succeeded in revealing the presence of at least 1 to 6 virulence determinants in the genomes of numerous isolates of *E. faecalis*, whereas other species were practically free of them. Jimenez et al. [87] scrutinized various strains of *Enterococcus* spp. isolated from animals and healthy humans and they found that isolates of *E. faecalis* contain several factors of virulence in their genome encompassing (*cad*, *ccf*, *cob*, *cpd*, *efa*A_fs_, *agg*, *gel*E, *cyl*A, *esp*), whereas *E. faecium* contained only the *efa*A_fm_ gene. In agreement with these findings, an investigation performed by Medeiros and his colleagues [198] in which they compared face-to-face strains isolated from the material of clinical origin versus strains from diverse food matrices including pasteurized milk, soft cheese raw meat and vegetables. They found that a number of strains isolated from foods and food products have similar virulence determinants in their genomes as those found in strains of clinical origin.

Numerous studies have corroborated that gelatinase represents the most frequently occurring factor of virulence [87,199,200]. Surprisingly, in 2014, Medeiros et al. [198] led a pilot research project in Brazil, in which they unveiled that the gene which encodes this metalloprotease has been detected in over 70% of isolates from food matrices (beetroot, cabbage, cassava, parsley, potato, sweet potato, raw meat, pasteurized milk and dairy products, including different types of cheese). Likewise, high levels of occurrence of *gel*E were spotted in enterococcal strains isolated from Turkish white cheese [201]. It is believed that frequent incidence of gelatinase ‘GelE’ in isolates from meat, meat derivatives and dairy products is mainly ascribed to the high content of these products in terms of gelatin, collagen and casein, which are substrates of gelatinase. This latter, as a factor of virulence, is often found in the genome of strains of clinical origin, encompassing vancomycin-resistant alarm pathogens. For instance, Sharifi et al. [196] succeeded in detecting the gelE gene in all vancomycin-resistant strains of clinical origin of *E. faecalis*. Similarly, Yılmaz et al. [202] identified the *gel*E gene in 75% of beef isolates and in 82.9% of chicken isolates.

Regarding the ace gene, its occurrence has been detected in enterococcal isolates of *E. faecalis* of food origin as well as in isolates of clinical origin [134,135]. In 2008, Abriouel et al. [203] demonstrated a higher occurrence of ace gene in clinical isolates, exceeding 80%, compared to isolates from other biotopes such as vegetables, water and soil.

On the list of alarming virulence determinants of enterococcal strains isolated from diverse foods and food products is the potential to form biofilm. This has led to a significant increase in the survival rate and propagation of antibiotic-resistance and the associated genes in a wide range of ecological niches and biotopes. Accordingly, bacterial strains, particularly enterococcal strains which live in the biofilm, have been shown to be more resistant to antibiotics [204]. It is pertinent to know that bacteria living within mature biofilm are able to tolerate a wide range of antibiotics at concentrations ranging from 10 to 1000 times higher than those living outside biofilm [205]. The presence of factors mediating adhesion to cells and formation of biofilm corresponds to the first main stage in the process of infection and/or colonization of a host. In the past, it was believed that there is a tight connection between the ability to form biofilm and the presence of *esp* [96,206]. However, another group of researchers reported that molecular mechanisms for biofilm formation are independent from *esp* [32,207,208].

Hancock and Perego [105] investigated the potential contribution of gelatinase in the process of biofilm formation. It was suspected that this contribution occurred via expediting signals arriving through the quorum-sensing *fsr* system. Such a hypothesis has been rejected by Mohamed and Murray [209] following the findings of their study where they unveiled a critical paucity of correlation between gelatinase and biofilm formation in a large collection of *E. faecalis* isolates. Earlier, Mohamed et al. [208] revealed the presence of a critical factor, the serine protease, which is even more important than gelatinase in biofilm production.

The hemolytic activity represents another critical virulence factor of enterococci isolated from food. In fact, Hammad et al. [210] succeeded in detecting the cylA gene related to cytolysin metabolism in enterococcal strains isolated from Egyptian fresh raw milk cheese. Likewise, *cyl*B has been detected in foodborne enterococcal strains, particularly isolated from Turkish white cheese [201]. It is important to note that numerous investigations have reported inconsistencies resulting from the presence of genes and their expression. Hence, the presence of cytolysin is not always associated with hemolysis on blood agar. In 2009, two pilot studies performed by Gaspar et al. [211] and Upadhyaya et al. [212] showed that the absence of hemolysis may result from a weak expression of the related gene, which does not allow the detection of phenotypic changes, or from the presence of an inactive gene product. In the same context, Trivedi et al. [112] demonstrated that β-hemolytic activity was higher in foodborne *E. faecalis* (29%) compared to *E. faecium* (10%) isolated from milk and dairy products, ready-to-eat meat products, fruits and vegetables. Accordingly, such a feature seems to be genus-dependent rather than species-dependent because it has been noted in *E. mundtii* and *E. durans* of dairy origin and in two *E. casseliflavus* strains of dairy and meat origin [112]. Despite all the findings mentioned above, diverse investigations have reported that Enterococcus strains isolated from a wide range of fermented food products exhibited no β-hemolytic activity [213,214].

Last but not least, sex pheromones as mediating factors in conjugation have been considered as a risk to food safety due to their high incidence in foodborne isolates. It is important to note that the high incidence of these factors in the genomes may be a good sign of their potential to exchange genetic material at every single stage of their production, and particularly when they reach the human GIT. It has been suggested that the incidence of sex pheromones is highly associated with the presence of the AS. Hence, enterococcal isolates that are endowed with the *asa*1 gene respond to the recipient’s cells via producing pheromones to accept the pheromone-dependent plasmids reflecting a crucial relationship in the process of exchange of resistance genetic determinants in the conjugation process. According to Akhtar et al. [215], the *ccf* gene is responsible for activating the conjugation of the pCF10 plasmid. This latter transfers resistance genes to tetracyclines. Furthermore, various researchers have shown that pheromones mediating in the system of conjugation are also responsible for acquiring resistance to glycopeptides, encompassing vancomycin. It is crucial to note that the transfer of the *van*A gene during conjugation by the method of membrane filters is highly facilitated by plasmid pCF10 [216,217]. Accordingly, sex pheromones seem to be a critical factor in the virulence processes of enterococcal strains particularly with their contribution in the spreading of antibiotic resistance. In 2010, Wardal and colleagues [218] led a pilot review investigation in which they demonstrated that epidemiological studies have shown that they are isolated more frequently from patients with bacteremia and wound infections than from feces samples from healthy volunteers and hospitalized patients. As mentioned above, the incidence of pheromones in foodborne isolates is a bit worrying especially in terms of their potential to mediate the process of gene exchange. For instance, numerous studies conducted on enterococcal strains (*n* = 35) isolated from retail raw (20 samples), cooked (20 samples), and ready-to-eat shrimps (20 samples) unveiled that genes encoding sex pheromones *cpd* (100%), *cob* (94.3%) and *ccf* (94.3%) were unexpectedly detected with high incidence. Similarly, in 2016, Chajecka-Wierzchowska et al. [219] succeeded in detecting the genetic determinants of sex pheromones, including *cpd*, *cob*, *ccf*, in 31 out of 35 enterococcal isolates from retail shrimps. At the same year, Yılmaz and coworkers [202] detected *cpd* (100% and 92.4%) and *ccf* (98% and 99%) as the most prevalent virulence determinants in enterococcal strains isolated from Turkish retail beef and chicken meat samples, respectively.

To sum up, it seems relevant to note the fact of the detection of virulence genetic determinants in the genome of various enterococci encompassing *E. casseliflavus*, *E. durans*, *E. hirae*, *E. avium*, *E. cecorum*, *E. gallinarum*, *E. malodoratus*, *E. faffinosus* and *E. mundtii* [86,201,220,221]. This is of outstanding importance considering the fact that scientific researchers usually focus on evaluating the virulence of *E. faecalis* and *E. faecium* as the most common representatives of *Enterococcus* spp. in food. In the meantime, the rise of other species containing invasiveness factors make them more virulent and sturdier.

## 7. Regulation of *Enterococcus* spp. Safety

In terms of safety issues, and according to the QPS list from the EFSA (https://www.efsa.europa.eu/en/topics/topic/qps, accessed on 1 September 2021), enterococcal species are neither recommended for the QPS list [222] nor have GRAS status [21], in spite of recent scientific knowledge allowing differentiation of commensal from pathogenic strains [223,224,225]. In this context, recent advancement in molecular epidemiology, particularly in terms of molecular fingerprinting, multi-locus sequence typing, phenotypic studies and whole-genome analyses, have delivered further evidence that Enterococcal nosocomial strains are genotypically different from commensal strains. In this regard, Montealegre et al. [223] unveiled, via molecular subtyping, the presence of three different clades of the *E. faecium*: A1 as the hospital-associated clade and rarely found in healthy individuals; A2 as the animal-associated clade; and the community-associated clade B, commonly found in healthy individuals and rarely causes infections. In the same context, in 2017, Beukers and collaborators [226] succeeded in comparing complete genomes of *E. faecium* from the NCBI database in order to disclose differential clustering of commensal and clinical isolates, indicating that these different *E. faecium* strains may be specifically adapted to their respective environments. Likewise, regarding *E. cecorum*, as a member of *Enterococcus* spp. genus, it has been reported that the difference between the ability of pathogenic and commensal *E. cecorum* isolates from different animal species to metabolize mannitol may be expounded by a separate evolution of pathogenic *E. cecorum* isolates [225]. In the same year, Bonacina et al. accurately investigated the genome sequences of four groups of *Enterococcus* species from food origin encompassing dairy, meat, probiotics and probiotics from dairy origin. They revealed the absence of any correlation between isolation source and/or probiotic properties and phylogenetic signal neither at species nor strain levels.

It is pertinent to mention that the remarkable progress outlined above endorses the urgent call for new recommendations in terms of probiotic regulation and legislative framework in order to discern between safe and potentially harmful Enterococcal strains. Leading organizations in food safety and security such as the European Food Safety Authority (EFSA), the Advisory Committee on Novel Foods and Processes (ACNFP), and the Food Standards Agency (FSA) allowed the use of certain strains of enterococci as a food additive and supplements based on a careful case-by-case appraisal. In this case, every single strain must be considered, and health risks must be excluded for this specific strain [39,227,228,229]. In husbandry, particularly in animal nutrition, the EFSA guidance [229], a globe reference in the field, delivers a meticulous and accurate procedure for distinguishing between safe and potentially harmful strains of *E. faecium*. For any kind of use as a feed additive microbial producer, applications might be submitted to EFSA for drastic safety evaluation. Regarding this international guidance, in order to be used in animal nutrition, enterococcal strains must be susceptible to ampicillin (MIC ≤ 2 mg/L) and must not harbor one of the genetic elements IS16, *hyl*E_fm_, and esp. In 2017, Brodmann and his team [230] showed that the full strain genome represents a mandatory benchmark of assessment of any new probiotic candidates by EFSA. To date, specific strains of *E. faecalis* and *E. faecium* are the only enterococci used as probiotics or feed additives [39]. The use of other enterococcal species is under contentious debate. It is subject to little or no regulation in spite of the steady number of investigations that unveil the probiotic potential of some species such as *E. munditii*, *E. durans* and *E. hirae* [19,231,232,233]. An array of recommended methods for the safety appraisal of *Enterococcus* is given in Table 4.

A wide range of bacteria showed a high potential to produce bioactive peptides, also known as ‘bacteriocins’. These bacteria are defined as proteinaceous compounds endowed with antimicrobial activity against Gram-positive and Gram-negative bacteria, and mainly produced by LAB which have a long history of application as natural food additives to enhance food safety [249,250]. In terms of legislative standards, nisin and pediocin PA1/AcH represent the only bacteriocins approved for utilization as food additives by the Food and Drug Administration (FDA) in USA and EU regardless the considerable number of experimental works on the application of other bacteriocins in food [251,252]. The use of LAB as microbial cell factories and their bacteriocins as an attractive alternative in the treatment of antibiotic-resistant bacterial infections seems promising, particularly with the current emergence of sturdy multi-resistant pathogens to conventional antibiotics [253]. Behrens et al. [254] reported the use of numerous bacteriocins in clinical trials and experiments. These proteinaceous compounds are well known by their potency, low toxicity and their specificity to target specific bacterial clusters reflecting a target accuracy without affecting much of the natural microbiota which is a common drawback of conventional antibiotic use [255,256]. According to Yang and coworkers [249], the gene-encoded nature of bacteriocins makes them easily amenable via bioengineering to either increase their activity or specify the target microorganism. However, probiotic strains or bacteriocins intended for use as a therapeutic must undergo the regulatory process as a new drug and must be authorized by the FDA [257]. It is also interesting to note that studies suggest that sub-lethal concentrations of bacteriocins such as nisin should have adverse effects by inducting an increase in virulence in surviving bacteria [258].

## 8. *Enterococcus* spp.: A Glance at Tomorrow—Focus on the Safety

In the strictest definition, “virulence factor” is defined as a substance that is necessary for causing disease in the host, but not necessary for survival in other contexts. For instance, *Bacillus anthracis*, a sturdy pathogenic bacterium which is able to produce toxins, mostly fits this criterion. While a significant number of genetic determinants contribute to the ability of a given enterococcal strain to cause infection, these factors are not necessarily found in every clinical isolate, which highlights the point that enterococcal infection is multifactorial and includes contributions by the microbe, as well as the host. Several enterococcal factors that contribute to fitness in the host also contribute to the overall fitness of the bacterium in other ecological biotopes, encompassing its normal habitat, the GIT. The virulence of enterococci is a more intricate phenomenon than the simple presence of some main players, and appears to be dependent on strain-variable combinations of factors that lead to improved infection and colonization when expressed together in the right background. Practically, all studies on enterococci as pathogens have focused on the genetic determinants that contribute to their sturdy pathogenic potential, with fewer studies focusing on the role of the host or on factors that contribute to the commensal lifestyle of enterococcal species. Promoting such noninfectious behavior could be a promising strategy for potentially preventing and treating infections. Understanding how enterococci contribute to the human microbiome as well as infection would help to illuminate exactly where they occur on the commensal–pathogen continuum. To summarize, these explanations represent a handful of concluding remarks that are meant to illustrate that we have only scratched the surface of this field.

Due to a poor understanding of adverse events arising in probiotic intervention investigations and a lack of structural and formal reporting of collateral effects, there remain uncertainties regarding probiotic safety. When looking at the large amount of data and scientific literature on probiotic safety showing that intake of probiotics is not associated with increased health risks, it is surprising that this is still a subject of debate. Though, there is one structural flaw in probiotic research that hampers making definitive conclusions regarding safety and harm [259]. It is pertinent to know that there is a structural under-reporting of adverse events and safety in terms of studies with probiotics, notably in clinical ones. This does not mean that the probiotic study design is poor in terms of quality, or the assessed probiotic strains are unsafe. Thus, to exemplify, as there is no data on the safety of eating apples, this does not mean that apples are unsafe to eat. Often no adverse effects are found during the study, or the experienced side effects are not worth mentioning. Researchers often suppose safety based on the long history of safety use or previous studies demonstrating no risks. Nevertheless, currently most probiotic intervention investigations are not equipped to correctly report adverse effects and properly classify them according to severity and relatedness. Additionally, experiments and scientific studies often fail to mention the investigated probiotic strain and its characteristics in terms of daily dosages, administration regimes, study populations and duration of the intervention. This is pivotal in purpose to establish the strain–dose–response relationship since effects are strain specific. For instance, it seemed difficult to extrapolate data to other strains reflecting an imminent limitation of the generalizability of concluding remarks.

To sum up, it is salient to note that probiotic investigators, even if they believe the probiotic strain is safe, adhere to the reporting of side effects. Thus, researchers contribute to developing a pertinent and accepted risk profile for diverse available probiotic strains that enable regulators and other stakeholders to make evidence-based accurate decisions. From our standpoint, generating adequate data on harms (or lack of harms) represents the only approach to overwhelm the current perceived uncertainties and barriers in the field of probiotic research. The lack of an obvious safety profile hampers innovation in each domain of the valorization cycle in terms of numerous elements: (*i*) scientific researchers are discouraged from conducting clinical experiments and trials in susceptible subjects; (*ii*) probiotic microorganisms are not being developed for these patients and are not recommended in the guidelines; (*iii*) health professionals such as physicians do not integrate probiotics in practice and (*iv*) a lack of lucidity in results leads to a lack of demand in probiotics.

## 9. Concluding Remarks and Future Outlook

*Enterococcus* spp., as a bacterial genus, belongs to the LAB group and is part of human-associated microbiota within diverse ecological niches encompassing skin, mouth, and GIT. As main features of numerous enterococcal strains, multi-bacteriocin production and their versatility to survive in different matrices including food and GIT, make them suitable candidates to fulfill the role as natural biopreservatives, as probiotics, or as viable alternatives to antibiotics. Moreover, enterococci bacteriocins, also known as enterocins, are recognized for their wide spectrum antagonism covering Gram-positive foodborne pathogens, such as biogenic amines producing bacteria [260], *L. monocytogenes* and Gram-negative bacteria. Furthermore, some bacteriocins exhibited antifungal and/or antiviral activity with an inhibitor potential of sporulating bacteria such as *Clostridium botulinum* and *Bacillus cereus* as well as an anti-endospores potency [261]. Outstandingly, these enterocins also displayed anti-cancer activities [262]. These attributes provide the justification to nominate bacteriocinogenic *Enterococcus* strains as relevant candidates for food, feed, human and animal health applications. In terms of competition amongst bacteria, bacteriocin production represents a key feature. Accordingly, bacteriocin-producing probiotics could compete with intestinal pathogens for colonization or modulate the microbiota homeostasis. Within this framework, two studies reported that bacteriocins can be produced in the gut by probiotic bacteria, where it can modulate gut microbiota to reduce gastrointestinal diseases [253,263].

Despite a long history of safe use in foods, the main question of whether enterococcal species can be used as starter, adjunct or probiotic cultures for the development of a new range of functional food products remains controversial, critical to answer and a difficult task to accomplish. Regarding the use of enterococci in foods, a critical issue emerges on the surface which is in direct correlation with their status as opportunistic pathogens, capable of causing infection in immunocompromised patients in nosocomial surroundings. The possession of putative virulence determinants along with an increasing prevalence of antibiotic resistance acquisition and transfer in some species, are hindering the progression of these microbes as important cultures in food technology applications. Nevertheless, the presence of virulence factors does not explicitly imply that the strains will cause imminent disease, but rather that they have pathogenic potential. For instance, virulence factors, directly related to adhesion and colonization, have been detected in the *E. faecalis* Symbioflor 1, a probiotic strain that has successfully been used for more than two decades without any reports of infection or as a causative agent of any kind of disease [61,264], reflecting the fact that the presence of virulence genes does not necessarily infer that they are functional. In the same context, it has been shown that within several enterococcal isolates carrying the *gel*E gene, gelatinase is not produced. Likewise, the ability to cause infection is considered a more intricate process than the possession of genetic virulence determinants in solo [39,63].

Several enterococcal members are firmly linked to severe healthcare-associated infections such as VRE, conferring a notorious reputation to the *Enterococcus* spp. genus. Hence, a major worry about use of enterococci strains in food supplements emerges, reflecting a potential spread of multi-antibiotic resistance and virulence genetic determinants [265]. Similarly, the alarming emergence of vancomycin resistance transfer from enterococci to methicillin-resistant strains of *Staphylococcus aureus* has been reported in more than one investigation [266]. Accordingly, bacterial strains carrying acquired resistance should not be deliberately introduced to the food and feed chain. 

At the scale of feed and novel food products, a pre-market safety assessment seems mandatory where the safety of the bacterial candidacy is drastically appraised at species-level. As previously mentioned, foodborne enterococcal strains harboring single or multiple virulence genes have been reported, even though, the case of ailments of healthy humans resulting from enterococcal infections seems to be very low [34,229,234]. In the context, Ogier and Serror [21] reported the absence of any kind of data showing a direct connection between the consumption of foods containing virulent enterococci and disease development. Of note, enterococcal isolates originating from food are usually susceptible to antibiotics of clinical relevance like ampicillin, penicillin, high-level aminoglycosides and the glycopeptides teicoplanin and vancomycin. Recent advances in molecular epidemiology and genomics have revealed that foodborne enterococcal strains are safe and the prevalence of virulence determinants is strain specific and that isolates from starter cultures harbor fewer virulence genes than food isolates. Thus, this latter possess fewer virulence factors than strains of clinical origin [223], reflecting a lower potential of pathogenicity [267]. This seems to be a promising tool to differentiate pathogenic from nonpathogenic enterococci and to enable improvement of the safety assessment of enterococci used in food and feed.

The ideal enterococcal probiotic would be bereft of all known virulence genes encompassing aspects for intestinal translocation of intact mucosal barriers, extracellular superoxide production, resistance to innate immunity, and resistance to phagocytic killing. With the era of Next-Generation Sequencing (NGS) technologies, these features are more easily evaluated in probiotic strains [44]. Additionally, probiotic strains should be completely impeded in their ability to exchange DNA although such a phenotype has yet to be created. In 2007, the European Food Safety Authority excluded enterococci from ‘QPS status’. Such a decision endorsed an evolving understanding of the pathogenesis of *E. faecalis* and *E. faecium* [30]. The potential use of other non-*E. faecalis* and non-*E. faecium* species as probiotics cannot be discounted although detailed studies into their virulence, resistance, and DNA exchange traits are required. To recap, enterococci, as opportunistic pathogens, are endowed with an arsenal of virulence factors and antibiotic resistance genes that render them poor choices as probiotics. The increasing number of nosocomial infections caused by bacteria, particularly by enterococci further enhance safety concerns and require more caution when using enterococcal probiotics. An accurate risk–benefit analysis along with relevant evidence for clinical efficacy are needed when these commensal microbes are to be used as probiotics.

In concluding the review, we must underscore the era of enterococcal genomics, a new direction that should drive future research in this field. Comparative genomics in enterococci has rapidly advanced over the last decade, and the number of genomes discussed in the scientific literature is constantly growing. For instance, 136 enterococcal genomes have been sequenced as part of the Human Microbiome Project (http://www.hmpdacc.org/, accessed on 15 October 2021), and 406 more were sequenced in a large-scale enterococcal genome sequencing endeavor performed in a multi-national collaboration with the Broad Institute (Cambridge, MA, USA). Obviously, our foray into enterococcal genomics has only just begun.

## Figures and Tables

**Table 1:** Selected enterococcal strains as probiotics for human consumption (adapted from [43] with modifications).

Commercial Product	Amount per Serving ^†^	Marketing and Uses ^¶^
***E. faecalis*** *		
Bifilac	30 million CFU per sachet or capsule	Treatment of diarrhea (traveler’s, antibiotic-associated, viral, bacterial, or protozoal); lactose intolerance; stomatitis; inflammatory bowel disease
Bioflora	n.d.	Restoration of flora following antibiotic treatment or chemotherapy; atrophic vaginitis; mild-to-moderate bacterial vaginosis and candidiasis
Pro-symbioflor	1.5–4.5 × 10^7^ CFU per 14 drops	Preparation of immune system for stomach and intestinal complaints
Shin-Biofermin S	2 mg per tablet	Intestinal regulation and treatment of diarrhea, constipation and meteorism
Symbioflor1	1.5–4.5 × 10^7^ CFU per 12 drops	Treatment of recurring sinusitis, bronchitis, pharyngitis; training of immune system
ThreeLac/FiveLac	500 million CFU	Elimination of symptoms of candidiasis; maintenance of intestinal health
Essential Formulas O’Hara Probiotic 12 plus OMX PROFESSIONAL Formula (*E. faecalis* TH10)	7.8–10 × 10^7^ CFU per capsule	Improvement of digestive and bowel function; decrease stomach disorders; treatment of Crohn’s disease; increase nutritional absorption; boosting appetite; decrease yeast infection; enhancement of liver health, circulatory, joint, and muscle function; improvement of sleep, vitamin synthesis, and resistance to allergies; lactose intolerance
***E. faecium*** *		
BIO-THREE (*E. faecium* ^§^ T-110)	n.d.	Homeostasis of intestinal microflora; inhibition of sturdy pathogens; facilitation of proliferation of *Bifidobacterium*; reduction in cholesterol; treatment of ulcerative colitis; prevention of colon cancer
N. American Herb & Spice Health-Bac Probiotic	n.d.	Support a healthy digestive response
Natural Factors Probiotics: *Acidophilus* with *E. faecium*	0.8 billion CFU per capsule	n.d.

* Only enterococcal species listed; products may contain other bacteria. Strains used cannot be identified except two products. ^†^ CFU, colony forming units. ^¶^ None of these claims or probiotics are approved by the U.S. FDA. ^§^ Although the manufacture claims it as *E. faecalis*, it is reported as an *E. faecium* [44]. n.d.: not defined.

**Table 2:** Examples of enterococcal probiotic formulas used in nutrition of livestock (adapted from [14]).

Commercial Name of the Formula (Manufacturer)	Microorganism(s) in the Preparation
**Poultry**	
B.I.O. Sol (Biochem)	*Enterococcus faecium*
Galvit Probiotyk (Galvit)	*Enterococcus faecium*
**Pigs**	
Anta Pro EF (Dr. Eckel)	*Enterococcus faecium*
Biogen T (Bio-Gen)	*Bifidobacterium bifidum*, *Lactobacillus acidophilus*, *Enterococcus faecium*
Cerbiopor	*Lactobacillus: Lactobacillus acidophilus*, *Levilactobacillus brevis*, *Lacticaseibacillus casei*, *Limosilactobacillus fermentum*, *Lactobacillus lactis*, *Lactiplantibacillus plantarum*; *Bacillus*: *subtilis*, *megaterium*, *pumilus*; *Enterococcus faecium*, *Cellulomonas* sp., *Saccharomyces cerevisiae*
**Cattle (calves)**	
Yea Sacc (Altech)	*Lacticaseibacillus rhamnosus, Enterococcus faecium*
**Poultry and pigs**	
Acid-Pak-4-Way (Alltech)	*Lactobacillus acidophilus, Enterococcus faecium*
Probios (Chr. Hansen)	*Lactobacillus*: *Lactobacillus acidophilus*, *Lacticaseibacillus casei*, *Lactiplantibacillus plantarum*, *lactis*; *Enterococcus faecium*; *Bacillus subtilis*
**Poultry and calves**	
Probiomix	*Bifidobacterium bifidum*, *Lactobacillus amylovorus*, *Enterococcus faecium*
UltraCruz (Santa Cruz Animal Health)	*Enterococcus faecium*, *Lactobacillus*: *Lactobacillus acidophilus*, *Lacticaseibacillus casei*, *Lactiplantibacillus plantarum*
**Calves and pigs**	
Cernivet LBC (Cerbios)	*Enterococcus faecium*
Provita LE (Schaumann)	*Lacticaseibacillus rhamnosus*, *Enterococcus faecium*
**Poultry, pigs, and calves**	
Cylactin (DSM)	*Enterococcus faecium*
Lactiferm	*Enterococcus faecium*
Oralin^®^ (Chevita GmbH)	*Enterococcus faecium*
**Poultry, pigs, sheep, and cattle**	
Protexin (Protexin Probiotics International Ltd.)	*Lactobacillus*: *Lactobacillus acidophilus*, *Lactobacillus delbruecki* subsp. *Bulgaricus*, *Lactiplantibacillus plantarum*, *Lacticaseibacillus rhamnosus*; *Bifidobacterium bifidum*; *Streptococcus salivarius* subsp. *Thermophilus*; *Enterococcus faecium*; *Aspergillus oryzae*; *Candida pintolepesii*
**Poultry, pigs, beef, dairy, horses, and deer**	
PrimaLac (Star Labs, Inc.)	*Bifidobacterium*: *bifidium*, *thermophilus*; *Enterococcus faecium*; *Lactobacillus*: *Lactobacillus acidophilus*, *Lacticaseibacillus casei*
**Poultry, pigs, pets, and livestock**	
SF68^®^ (Cerbios-Pharma SA)	*Enterococcus faecium*
Symbioflor^®^ 1 (Symbiopharm, Herborn)	*Enterococcus faecalis*

**Table 3:** Enterococcal virulence determinants and their putative role.

Enterococcal Virulence Factors	Gene(s)	Reported Biological Effect
**Virulence factors that promote colonization**		
Aggregation substance (AS)	*agg, prg*B *asa*1	Binding to host cells, enables cell-to-cell contact between donor and recipient strains for conjugation; facilitating binding of donor and recipient cells; to mediate adhesion of *E. faecalis* to eukaryotic cells and internalization; enhancement of bacterial vegetation; synergetic regulation of quorum via Cyl; protection against neutrophilic killing
Collagen-binding protein (Ace) Microbial surface components recognizing adhesive matrix molecules (MSCRAMMs)	*ace* *acm*, *fss1*, *fss2* and *fss3*	Colonization by binding to proteins of the extracellular matrix (ECM); participation in binding type I and IV host collagen; adherence to host fibronectin, fibrinogen, and/or laminin
Cell wall adhesin (Efa A)	*efa*A	Virulence factors associated with infective endocarditis
Enterococcal surface protein (Esp)	*esp*	Surface adhesion, colonization, and persistence; association with biofilm formation
Extracellular superoxide	*men*A, *men*B, *cyd*A, and *frd*A	Bacterial invasion; clastogen and mutagen
Pilus	*ebp* (A–C), *srtA*, *bps* (*srt*C), and *bee* locus	Adherence to host cells; enhances biofilm formation
**Virulence factors with affect tissues**		
Cytolysin (*Cyl*),	*cyl*L_L_*, cyl*L_S_*, cyl*M*, cyl*B, *cyl*A*, cyl*I*, cyl*R1, and *cyl*R2	Bactericidal properties towards Gram-negative bacteria; toxic properties (β-hemolysis) towards mammalian erythrocytes (not sheep or goat), leukocytes, macrophages; destroys neural tissues; bacteriocin; quorum regulation
Gelatinase (*Gel*E)	*efa*A_fs_, *efa*A_fm_, *gel*E, *spr*E, *fsr*A, *fsr*B, and *fsr*C	Hydrolysis of gelatin, elastin, collagen, hemoglobin, polymerized fibrin as well as other bioactive peptides, e.g., proteins bound to pheromones; cleavage of human complement C3 and C5a; clearing misfolded bacterial surface proteins and extracellular pheromones; maintaining diplococcal morphology
Hyaluronidase (*Hyl*)	*hyl*A	Playing a key role in destroying mucopolysaccharides of the connective tissue and cartilage

**Table 4:** Recommended methods for the safety appraisal of *Enterococcus* (non-QPS species) (adapted from [25]) with slight modification.

Experimental Trials	Approach	References
**Antibiotic susceptibility**		
Phenotypic antibiotic susceptibility: Ampicillin	Minimal inhibitory concentrations (MICs) (mg/L or µg/mL; susceptibility testing: EUCAST/CLSI, ISO standard)	[229]
**Other Considerations**		
Susceptibility to clinically relevant antibiotics: (vancomycin, gentamicin, kanamycin, streptomycin, erythromycin, clindamycin, tetracycline, chloramphenicol)	Minimal inhibitory concentrations (MICs) (mg/L or µg/mL; susceptibility testing: EUCAST/CLSI, ISO standard)	[234,235]
**Detection of Virulence Markers Associated with Clinical Strains**		
*esp*	Hybridization techniques	[236]
*hyl*E_fm_	PCR	[237]
*IS*16	PCR	[238]
	Alternative methods: - Hybridization to colony lysates - Southern blots	[239]
**Other Considerations**		
*Genotypic assessment*	- Multilocus sequence typing (MLST) - DNA fingerprint - PCR	
**Vancomycin operons** (*van*A, *van*B, *van*C, *van*D, *van*E, *van*G, *van*M, *van*L, *van*N)		[240]
**Aggregation protein gene** (agg)		
**Surface adhesin genes** (*efa*A_fs_, *efa*A_fm_)		[82]
**Cytolysin genes** (*cyl*L_L_, *cyl*L_s_, *cyl*M, *cyl*B, *cyl*A)		
**Extracellular metalloendopeptidase***gel*E		[241]
*Phenotypic assessment*		
**Hemolytic Activity**	Hemolytic activity assay on 5% sheep or horse blood Columbia agar plates	[242]
**Gelatin hydrolysis**	Assay for gelatinase activity on Todd-Hewitt (TH) agar plates containing 3% gelatin	[243]
**Biogenic Amines Detection**		
Histamine Putrescine Phenylethylamine Cadaverine	High pressure liquid chromatography (HPLC)	[244]
	Alternative methods (for Histamine)	
	- Fluorometric methods - Immunoassays - Flow injection analysis - Colorimetric method	[245,246,247]
**Detection of amino acid decarboxylase-positive** **microorganisms**	- Quantitative real-time PCR histamine-producing LAB - In vitro detection method (Enzymatic or chemical analysis)	[244,245,248]
**Toxin Production**		
Cytotoxic potential	Vero cell cytotoxicity test	[235]
Full genome (*When available*)	Next Generation Sequencing	[229]

## References

[1] FAO/WHO (2002). Guidelines for the Evaluation of Probiotics in Food.

[2] Hill C., Guarner F., Reid G., Gibson G.R., Merenstein D.J., Pot B., Morelli L., Canani R.B., Flint H.J., Salminen S. (2014). The International Scientific Association for Probiotics and Prebiotics consensus statement on the scope and appropriate use of the term probiotic. Nat. Rev. Gastroenterol. Hepatol..

[3] Zendeboodi F., Khorshidian N., Mortazavian A.M., da Cruz A.G. (2020). Probiotic: Conceptualization from a new approach. Curr. Opin. Food Sci..

[4] Zommiti M., Connil N., Ben Hamida J., Ferchichi M. (2017). Probiotic Characteristics of *Lactobacillus curvatus* DN317, a Strain Isolated from Chicken Ceca. Probiotics Antimicrob. Proteins.

[5] Zommiti M., Cambronel M., Maillot O., Barreau M., Sebei K., Feuilloley M., Ferchichi M., Connil N. (2018). Evaluation of Probiotic Properties and Safety of *Enterococcus faecium* Isolated From Artisanal Tunisian Meat “Dried Ossban”. Front. Microbiol..

[6] Zommiti M., Bouffartigues E., Maillot O., Barreau M., Szunerits S., Sebei K., Feuilloley M., Connil N., Ferchichi M. (2018). *In vitro* Assessment of the Probiotic Properties and Bacteriocinogenic Potential of *Pediococcus pentosaceus* MZF16 Isolated From Artisanal Tunisian Meat “Dried Ossban”. Front. Microbiol..

[7] Daba G.M., El-Dien A.N., Saleh S.A.A., Elkhateeb W.A., Awad G., Nomiyama T., Yamashiro K., Zendo T. (2021). Evaluation of *Enterococcus* strains newly isolated from Egyptian sources for bacteriocin production and probiotic potential. Biocatalysis Agric. Biotechnol..

[8] Fugaban J.I.I., Holzapfel W.H., Todorov S.D. (2021). Probiotic potential and safety assessment of bacteriocinogenic *Enterococcus faecium* strains with antibacterial activity against *Listeria* and vancomycin-resistant enterococci. Curr. Res. Microb. Sci..

[9] Hamid N.H., Daud H.M., Kayansamruaj P., Hassim H.A., Yusoff M.S.M., Abu Bakar S.N., Srisapoome P. (2021). Short- and long-term probiotic effects of *Enterococcus hirae* isolated from fermented vegetable wastes on the growth, immune responses, and disease resistance of hybrid catfish (*Clarias gariepinus* × *Clarias macrocephalus*). Fish Shellfish Immunol..

[10] Oruc O., Cetin O., Darilmaz D.O., Yüsekdag Z.N. (2021). Determination of the biosafety of potential probiotic *Enterococcus faecalis* and *Enterococcus faecium* strains isolated from traditional white cheeses. LWT Food Sci. Technol..

[11] Markowiak, P, Śliżewska, K (2017). Effects of probiotics, prebiotics, and synbiotics on human health. Nutrients.

[12] Goderska K., Agudo Pena S., Alarcon T. (2018). *Helicobacter pylori* treatment: Antibiotics or probiotics. Appl. Microbiol. Biotechnol..

[13] Casas-Solís J., Huizar-López M.D.R., Irecta-Nájera C.A., Pita-López M.L., Santerre A. (2019). Immunomodulatory effect of *Lactobacillus casei* in a murine model of colon carcinogenesis. Probiotics Antimicrob. Proteins.

[14] Zommiti M., Chikindas M.L., Ferchichi M. (2019). Probiotics-Live Biotherapeutics: A Story of Success, Limitations, and Future Prospects-Not Only for Humans. Probiotics Antimicrob. Proteins.

[15] Zommiti M., Feuilloley M.G.J., Connil N. (2020). Update of Probiotics in Human World: A Nonstop Source of Benefactions till the End of Time. Microorganisms.

[16] Uymaz Tezel B. (2019). Preliminary *In Vitro* Evaluation of the Probiotic Potential of the Bacteriocinogenic Strain *Enterococcus lactis* PMD74 Isolated from Ezine Cheese. J. Food Qual..

[17] Miller W.R., Murray B.E., Rice L.B., Arias C.A. (2020). Resistance in Vancomycin-Resistant Enterococci. Infect. Dis. Clin. N. Am..

[18] Rehaiem A., Belgacem Z.B., Edalatian M.R., Martínez B., Rodríguez A., Manai M., Guerrae N.P. (2014). Assessment of potential probiotic properties and multiple bacteriocin encoding-genes of the technological performing strain *Enterococcus faecium* MMRA. Food Control.

[19] Pieniz S., Andreazza R., Anghinoni T., Camargo F., Brandelli A. (2014). Probiotic potential, antimicrobial and antioxidant activities of *Enterococcus durans* strain LAB18s. Food Control.

[20] Giraffa G. (2003). Functionality of enterococci in dairy products. Int. J. Food Microbiol..

[21] Ogier J.C., Serror P. (2008). Safety assessment of dairy microorganisms: The *Enterococcus* genus. Int. J. Food Microbiol..

[22] Wu Y., Renxian, Li R., Ding W., Croes J., Yang M. (2019). Mechanism Study and Reduction of Minivan Interior Booming Noise during Acceleration. Shock Vib..

[23] Tachibana L., Telli G.S., de Carla Dias D., Gonçalves G.S., Ishikawa C.M., Cavalcante R.B., Natori M.M., BenHamed S., Ranzani-Paiva M.J.T. (2020). Effect of feeding strategy of probiotic *Enterococcus faecium* on growth performance, hematologic, biochemical parameters and non-specific immune response of Nile tilapia. Aquacult. Rep..

[24] Furlaneto-Maia L., Ramalho R., Rocha K.R., Furlaneto M.C. (2020). Antimicrobial activity of enterocins against *Listeria* sp. and other food spoilage bacteria. Biotechnol. Lett..

[25] Hanchi H., Mottawea W., Sebei K., Hammami R. (2018). The genus *Enterococcus*: Between probiotic potential and safety concerns-an update. Front. Microbiol..

[26] Nami Y., Bakhshayesh R.V., Jalaly H.M., Lotfi H., Eslami S., Hejazi M.A. (2019). Probiotic properties of *Enterococcus* isolated from artisanal dairy products. Front. Microbiol..

[27] Pajarilloa E.A.B., Chae J.P., Balolong M.P., Kim H.B., Park C.S., Kang D.K. (2015). Effects of probiotic *Enterococcus faecium* NCIMB 11181 administration on swine fecal microbiota diversity and composition using barcoded pyrosequencing. Anim. Feed Sci. Technol..

[28] Holzapfel W., Arini A., Aeschbacher M., Coppolecchia R., Pot B. (2018). *Enterococcus faecium* SF68 as a model for efficacy and safety evaluation of pharmaceutical probiotics. Benef. Microbes.

[29] Morandi S., Silvetti T., Brasca M. (2013). Biotechnological and safety characterization of *Enterococcus lactis*, a recently described species of dairy origin. Antonie Van Leeuwenhoek.

[30] Barlow S., Chesson A., Collins J.D., Dybing E., Flynn A., Fruijtier-Pölloth C., Hardy A., Knaap A., Kuiper H., Le Neindre P. (2007). Introduction of a qualified presumption of safety (QPS) approach for assessment of selected microorganisms referred to EFSA. EFSA J..

[31] Arias C.A., Murray B.E. (2012). The rise of the *Enterococcus*: Beyond vancomycin resistance. Nat. Rev. Microbiol..

[32] Kristich C.J., Li Y.H., Cvitkovitch D.G., Dunny G.M. (2004). *Esp*-independent biofilm formation by *Enterococcus faecalis*. J. Bacteriol..

[33] Fiore E., van Tyne D., Gilmore M.S. (2019). Pathogenicity of Enterococci. Microbiol Spectr..

[34] Franz C.M., Stiles M.E., Schleifer K.H., Holzapfel W.H. (2003). Enterococci in foods-a conundrum for food safety. Int. J. Food Microbiol..

[35] Busani L., Del Grosso M., Paladini C., Graziani C., Pantosti A., Biavasco F., Caprioli A. (2004). Antimicrobial susceptibility of vancomycin-susceptible and resistant enterococci isolated in Italy from raw meat products, farm animals, and human infections. Int. J. Food Microbiol..

[36] Willems R.J., Bonten M.J.M. (2007). Glycopeptide-resistant enterococci: Deciphering virulence, resistance and epidemicity. Curr. Opin. Infect. Dis..

[37] Inoglu Z.N., Tuncer Y. (2013). Safety assessment of *Enterococcus faecium* and *Enterococcus faecalis* strains isolated from Turkish Tulum cheese. J. Food Saf..

[38] Chiang H.Y., Perencevich E.N., Nair R., Nelson R.E., Samore M., Khader K., Chorazy M.L., Herwaldt L.A., Blevins A., Ward M.A. (2017). Incidence and outcomes associated with infections caused by vancomycin-resistant enterococci in the United States: Systematic literature review and meta-analysis. Infect. Control Hosp. Epidemiol..

[39] Franz C.M.A.P., Huch M., Abriouel H., Holzapfel W., Gálvez A. (2011). *Enterococci* as probiotics and their implications in food safety. Int. J. Food Microbiol..

[40] Agudelo Higuita N.I., Huycke M.M., Gilmore M.S., Clewell D.B., Ike Y., Shankar N. (2014). Enterococcal Disease, Epidemiology, and Implications for Treatment. Enterococci: From Commensals to Leading Causes of Drug Resistant Infection.

[41] Nascimento L.C., Casarotti S.N., Todorov S.D., Penna A.L. (2019). Probiotic potential and safety of enterococci strains. Ann. Microbiol..

[42] Santos Melo C.C., Freire A.S., Galdeano M.A., da Costa C.F., Gonçalves A.P.D.O., Dias F.S., Menezes D.R. (2021). Probiotic potential of *Enterococcus hirae* in goat milk and its survival in canine gastrointestinal conditions simulated *in vitro*. Res. Vet. Sci..

[43] Wang X., Yang Y., Huycke M.M. (2020). Risks associated with enterococci as probiotics. Food Res. Int..

[44] Natarajan P., Parani M. (2015). First complete genome sequence of a probiotic *Enterococcus faecium* strain T-110 and its comparative genome analysis with pathogenic and non-pathogenic *Enterococcus faecium* genomes. J. Genet. Genom..

[45] Economou V., Sakkas H., Delis G., Gousia P., Singh O.V. (2017). Antibiotic resistance in *Enterococcus* spp. friend or foe?. Foodborne Pathogens and Antibiotic Resistance.

[46] Olvera-Garcıa M., Sanchez-Flores A., Quirasco Baruch M. (2018). Genomic and functional characterisation of two *Enterococcus* strains isolated from Cotija cheese and their potential role in ripening. Appl. Microbiol. Biotechnol..

[47] Bybee S., Scorza A., Lappin M. (2011). Effect of the probiotic *Enterococcus faecium* SF68 on presence of diarrhea in cats and dogs housed in an animal shelter. J. Vet. Intern. Med..

[48] Opera S.F., Zervos M.J. (2007). Enterococcus and its association with foodborne illness. Infectious Disease: Foodborne Diseases.

[49] Riemann H., Bryan F.L. (1979). Foodborne Infections and Intoxications.

[50] Guerrero-Ramos E., Cordero J., Molina-Gonzalez D., Poeta P., Igrejas G., Alonso-Calleja C., Capita R. (2016). Antimicrobial resistance and virulence genes in enterococci from wild game meat in Spain. Food Microbiol..

[51] Baccouri O., Boukerb A.M., Farhat L.B., Zébré A., Zimmermann K., Domann E., Cambronel M., Barreau M., Maillot O., Rincé I. (2019). Probiotic Potential and Safety Evaluation of *Enterococcus faecalis* OB14 and OB15, Isolated From Traditional Tunisian Testouri Cheese and Rigouta, Using Physiological and Genomic Analysis. Front. Microbiol..

[52] Tomita H., Ike Y. (2004). Tissue-specific adherent *Enterococcus faecalis* strains that show highly efficient adhesion to human bladder carcinoma T24 cells also adhere to extracellular matrix proteins. Infect. Immun..

[53] Foulquie Moreno M.R., Sarantinopoulos P., Tsakalidou E., De Vuyst L. (2006). The role and application of enterococci in food and health. Int. J. Food Microbiol..

[54] Strzelecki J., Sadowy E., Hryniewicz W. (2011). Enterococcal surface proteins responsible for interactions with host tissues. Adv. Microbiol..

[55] Hollenbeck B., Rice L.B. (2012). Intrinsic and acquired resistance mechanisms in enterococcus. Virulence.

[56] Gentry-Weeks C.R., Karkhoff-Schweizer R., Pikis A., Estay M., Keith J.M. (1999). Survival of *Enterococcus faecalis* in mouse peritoneal macrophages. Infect. Immun..

[57] Huycke M.M., Moore D., Joyce W., Wise P., Shepard L., Kotake Y., Gilmore M.S. (2001). Extracellular superoxide production by *Enterococcus faecalis* requires de-methylmenaquinone and is attenuated by functional terminal quinol oxidases. Mol. Microbiol..

[58] Wang X., Huycke M.M. (2007). Extracellular superoxide production by *Enterococcus faecalis* promotes chromosomal instability in mammalian cells. Gastroenterology.

[59] Wang X., Allen T.D., May R.J., Lightfoot S., Houchen C.W., Huycke M.M. (2008). *Enterococcus faecalis* induces aneuploidy and tetraploidy in colonic epithelial cells through a bystander effect. Cancer Res..

[60] Wang X., Yang Y., Moore D.R., Nimmo S.L., Lightfoot S.A., Huycke M.M. (2012). 4-Hydroxy-2-nonenal mediates genotoxicity and bystander effects caused by *Enterococcus faecalis*-infected macrophages. Gastroenterology.

[61] Domann E., Hain T., Ghai R., Billion A., Kuenne C., Zimmermann K., Chakraborty T. (2007). Comparative genomic analysis for the presence of potential enterococcal virulence factors in the probiotic *Enterococcus faecalis* strain Symbioflor 1. Int. J. Med. Microbiol..

[62] Pillar C.M., Gilmore M.S. (2004). Enterococcal virulence–pathogenicity island of *E. faecalis*. Front. Biosci..

[63] Chajecka-Wierzchowska W., Zadernowska A., Łaniewska-Trokenheim Ł. (2017). Virulence factors of *Enterococcus* spp. presented in food. LWT—Food Sci. Technol..

[64] Ben Braiek O., Smaoui S. (2019). *Enterococci*: Between Emerging Pathogens and Potential Probiotics. BioMed Res. Int..

[65] Garsin D.A., Frank K.L., Silanpää J., Ausubel F.M., Hartke A., Shankar N., Murray B.E., Gilmore M.S., Clewell D.B., Ike Y., Shankar N. (2014). Pathogenesis and Models of Enterococcal Infection. Enterococci: From Commensals to Leading Causes of Drug Resistant Infection.

[66] Paulsen I.T., Banerjei L., Myers G.S., Nelson K.E., Seshadri R., Read T.D., Fouts D.E., Eisen J.A., Gill J.F., Heidelberg S.R. (2003). Role of mobile DNA in the evolution of vancomycin-resistant *Enterococcus faecalis*. Science.

[67] Hirt H., Manias D.A., Bryan E.M., Klein J.R., Marklund J.K., Staddon J.H., Paustian M.L., Kapur V., Dunny G.M. (2005). Characterization of the pheromone response of the *Enterococcus faecalis* conjugative plasmid pCF10: Complete sequence and comparative analysis of the transcriptional and phenotypic responses of pCF10-containing cells to pheromone induction. J. Bacteriol..

[68] Bhatty M., Cruz M.R., Frank K.L., Gomez J.A., Andrade F., Garsin D.A., Dunny G.M., Kaplan H.B., Christie P.J. (2015). *Enterococcus faecalis* pCF10-encoded surface proteins PrgA, PrgB (aggregation substance) and PrgC contribute to plasmid transfer, biofilm formation and virulence. Mol. Microbiol..

[69] Dramsi S., Trieu-Cuot P., Bierne H. (2005). Sorting sortases: A nomenclature proposal for the various sortases of gram-positive bacteria. Res. Microbiol..

[70] Schmitt A., Jiang K., Camacho M.I., Jonna V.R., Hofer A., Westerlund F., Christie P.J., Berntsson R.P. (2018). PrgB promotes aggregation, biofilm formation, and conjugation through DNA binding and compaction. Mol. Microbiol..

[71] Schlievert P.M., Gahr P.J., Assimacopoulos A.P., Dinges M.M., Stoehr J.A., Harmala J.W., Hirt H., Dunny G.M. (1998). Aggregation and binding substances enhance pathogenicity in rabbit models of *Enterococcus faecalis* endocarditis. Infect. Immun..

[72] Chuang O.N., Schlievert P.M., Wells C.L., Manias D.A., Tripp T.J., Dunny G.M. (2009). Multiple functional domains of *Enterococcus faecalis* aggregation substance Asc10 contribute to endocarditis virulence. Infect. Immun..

[73] Clewell D.B., An F.Y., Flannagan S.E., Antiporta M., Dunny G.M. (2000). Enterococcal sex pheromone precursors are part of signal sequences for surface lipoproteins. Mol. Microbiol..

[74] Dunny G.M., Leonard B.A., Hedberg P.J. (1995). Pheromone-inducible conjugation in *Enterococcus faecalis*: Interbacterial and host—Parasite chemical communication. J. Bacteriol..

[75] Kozlowicz B.K., Dworkin M., Dunny G.M. (2006). Pheromone inducible conjugation in *Enterococcus faecalis*: A model for the evolution of biological complexity?. Int. J. Med. Microbiol..

[76] Wardal E., Gawryszewska I., Hryniewicz W., Sadowy E. (2013). Abundance and diversity of plasmid-associated genes among clinical isolates of *Enterococcus faecalis*. Plasmid.

[77] Gilmore S.M., Coburn P.S., Nallapareddy S.R., Murray B.E., Gilmore M.S. (2002). Enterococcal virulence. The Enterococci: Pathogenesis, Molecular Biology, and Antibiotic Resistance.

[78] Clewell D.B. (2007). Properties of *Enterococcus faecalis* plasmid pAD1, a member of a widely disseminated family of pheromone-responding, conjugative, virulence elements encoding cytolysin. Plasmid.

[79] Dunny G.M. (2007). The peptide pheromone-inducible conjugation system of *Enterococcus faecalis* plasmid pCF10: Cell-cell signalling, gene transfer, complexity and evolution. Philos. Trans. R. Soc. Lond. B Biol. Sci..

[80] Hendrickx A.P., Willems R.J., Bonten M.J., Van Schaik W. (2009). LPxTG surface proteins of enterococci. Trends Microbiol..

[81] Suk-Kyung L., Koichi T., Haruyoshi T., Yasuyoshi I. (2006). Pheromone- Responsive conjugative vancomycin resistance plasmids in *Enterococcus faecalis* isolates from humans and chicken feces. Appl. Environ. Microbiol..

[82] Eaton T.J., Gasson M.J. (2001). Molecular screening of *Enterococcus* virulence determinants and potential for genetic exchange between food and medical isolates. Appl. Environ. Microbiol..

[83] Sava I.G., Heikens E., Huebner J. (2010). Pathogenesis and immunity in enterococcal infections. Clin. Microbiol. Infect..

[84] Abrantes M.C., Kok J., Lopes Mde F. (2013). EfaR is a major regulator of *Enterococcus faecalis* manganese transporters and influences processes involved in host colonization and infection. Infect. Immun..

[85] Archimbaud C., Shankar N., Forestier C., Baghdayan A., Gilmore M.S., Charbonné F., Joly B. (2002). *In vitro* adhesive properties and virulence factors of *Enterococcus faecalis* strains. Res. Microbiol..

[86] Semedo T., Santos M.A., Lopes M.F., Marques J.J.F., Crespo M.T., Tenreiro R. (2003). Virulence factors in food, clinical and reference enterococci: A common trait in the genus?. Syst. Appl. Microbiol..

[87] Jimenez E., Ladero V., Chico I., Maldonado-Barragan A., Lopez M., Martín V., Fernández L., Fernández M., Álvarez M.A., Torres C. (2013). Antibiotic resistance, virulence determinants and production of biogenic amines among enterococci from ovine, feline, canine, porcine and human milk. BMC Microbiol..

[88] Shankar N., Baghdayan A.S., Gilmore M.S. (2002). Modulation of virulence within a pathogenicity island in vancomycin-resistant *Enterococcus faecalis*. Nature.

[89] Leavis H., Top J., Shankar N., Borgen K., Bonten M., van Embden J., Willems R.J.L. (2004). A novel putative pathogenicity Island linked to esp virulence gene of *Enterococcus faecium* and associated with epidemicity. J. Bacteriol..

[90] Shankar V., Baghdayan A.S., Huycke M.M., Lindahl G., Gilmore M.S. (1999). Infection derived *Enterococcus faecalis* strains are enriched in esp, a gene encoding a novel surface protein. Infect. Immun..

[91] Shankar N., Lockatell C.V., Baghdayan A.S., Drachenberg C., Gilmore M.S., Johnson D.E. (2001). Role of *Enterococcus faecalis* surface protein Esp in the pathogenesis of ascending urinary tract infection. Infect. Immun..

[92] Top J., Paganelli F.L., Zhang X., van Schaik W., Leavis H.L., van Luit-Asbroek M., Willems R.J. (2013). The *Enterococcus faecium* enterococcal biofilm regulator, EbrB, regulates the esp operon and is implicated in biofilm formation and intestinal colonization. PLoS ONE.

[93] Heikens E., Singh K.V., Jacques-Palaz K.D., van Luit-Asbroek M., Oostdijk E.A.N., Bonten M.J.M., Murray B.E., Willems R.J.L. (2011). Contribution of the enterococcal surface protein Esp to pathogenesis of *Enterococcus faecium* endocarditis. Microbes Infect..

[94] Toledo-Arana A., Valle J., Solano C., Arrizubieta M.J., Cucarella C., Lamata M., Amorena B., Leiva J., Penadés J.R., Lasa I. (2001). The enterococcal protein, Esp, is involved in *Enterococcus faecalis* biofilm formation. Appl. Environ. Microbiol..

[95] Donlan R.M., Costerton J.W. (2002). Biofilms: Survival mechanisms of clinically relevant microorganisms. Clin. Microbiol. Rev..

[96] Tendolkar P.M., Baghdayan A.S., Gilmore M.S., Shankar N. (2004). Enterococcal surface protein, Esp, enhances biofilm formation by *Enterococcus faecalis*. Infect. Immun..

[97] Latasa C., Solano C., Penade S.J.R., Lasa I. (2006). Biofilm associated proteins. Comptes Rendus Biol..

[98] Billstrom H., Lund B., Sullivan A., Nord C.E. (2008). Virulence and antimicrobial resistance in clinical *Enterococcus faecium*. Int. J. Antimicrob. Agents.

[99] Ochoa S.A., Escalona G., Cruz-Cordova A., Davila L.B., Saldana Z., Cazares-Domímguez V., Eslava C.A., López-Martínez B., Hernández-Castro R., Aquino-Jarquin G. (2013). Molecular analysis and distribution of multidrug—resistant *Enterococcus faecium* isolates belonging to clonal complex 17 in a tertiary care center in Mexico City. BMC Microbiol..

[100] Oancea C., Klare I., Witte W., Werner G. (2004). Conjugative transfer of the virulence gene, *esp*, among isolates of *Enterococcus faecium* and *Enterococcus faecalis*. J. Antimicrob. Chem..

[101] Goguen J.D., Hoe N.P., Subrahmanyam Y.V. (1995). Proteases and bacterial virulence: A view from the trenches. Infect. Agents Dis..

[102] Park S.Y., Shin Y.P., Kim C.H., Park H.J., Seong Y.S., Kim B.S., Seo S.J., Lee I.H. (2008). Immune evasion of *Enterococcus faecalis* by an extracellular gelatinase that cleaves C3 and iC3b. J. Immunol..

[103] Maharshak N., Huh E.Y., Paiboonrungruang C., Shanahan M., Thurlow L., Herzog J., Djukic Z., Orlando R., Pawlinski R., Ellermann M. (2015). *Enterococcus faecalis* gelatinase mediates intestinal permeability via protease-activated receptor 2. Infect. Immun..

[104] Waters C.M., Antiporta M.H., Murray B.E., Dunny G.M. (2003). Role of the *Enterococcus faecalis* GelE protease in determination of cellular chain length, supernatant pheromone levels, and degradation of fibrin and misfolded surface proteins. J. Bacteriol..

[105] Hancock L.E., Perego M. (2004). The *Enterococcus faecalis* fsr two-component system controls biofilm development through production of gelatinase. J. Bacteriol..

[106] Pillai S.K., Sakoulas G., Eliopoulos G.M., Moellering R.C., Murray B.E., Inouye R.T. (2004). Effects of glucose on fsr-mediated biofilm formation in *Enterococcus faecalis*. J. Infect. Dis..

[107] Podbielski A., Kreikemeyer B. (2004). Cell density-dependent regulation, basic principles and effects on the virulence of Gram-positive cocci. Int. J. Infect. Dis..

[108] Mohamed J.A., Huang D.B. (2007). Biofilm formation by enterococci. J. Med. Microbiol..

[109] Pinkston K.L., Gao P., Diaz-Garcia D., Sillanpa J., Nallapareddy S.R., Murray B.E., Harvey B.R. (2011). The Fsr quorum-sensing system of *Enterococcus faecalis* modulates surface display of the collagen-binding MSCRAMM Ace through regulation of gelE. J. Bacteriol..

[110] Teixeira N., Santos S., Marujo P., Yokohata R., Iyer V.S., Nakayama J., Hancock L.E., Serror P., Lopes M.F.S. (2012). The incongruent gelatinase genotype and phenotype in *Enterococcus faecalis* are due to shutting off the ability to respond to the gelatinase biosynthesis-activating pheromone (GBAP) quorum-sensing signal. Microbiology.

[111] Vankerckhoven V., Van Autgaerden T., Vael C., Lammens C., Chapelle S., Rossi R., Jabes D., Goossens H. (2004). Development of Multiplex PCR for the detection of *asa1*, *gelE*, *cylA*, *esp*, and *hyl* genes in Enterococci and survey for virulence determinants among European hospital isolates of *Enterococcus faecium*. J. Clin. Microbiol..

[112] Trivedi K., Cupakova S., Karpiskova R. (2011). Virulence factors and antibiotic resistance in Enterococci isolated from food-stuffs. Vet. Med..

[113] Comerlato C.B., Resende M.C., Caierao J., D’azevedo P.A. (2013). Presence of virulence factors in *Enterococcus faecalis* and *Enterococcus faecium* susceptible and resistant to vancomycin. Mem. Inst. Oswaldo Cruz.

[114] Huddleston J.R. (2014). Horizontal gene transfer in the human gastrointestinal tract: Potential spread of antibiotic resistance genes. Infect. Drug Resis..

[115] Franz C.M.A.P., Muscholl-Silberhorn A.B., Yousif N.M.K., Vancanneyt M., Swings J., Holzapfel W.H. (2001). Incidence of virulence factors and antibiotic resistance among enterococci isolated from food. Appl. Environ. Microbiol..

[116] Giraffa G. (2002). Enterococci from foods. FEMS Microbiol. Rev..

[117] Clewell D.B., Francia M.V., Flannagan S.E., An F.Y. (2002). Enterococcal plasmid transfer: Sex pheromones, transfer origins, relaxases, and the *Staphylococcus aureus* issue. Plasmid.

[118] Chandler J.R., Dunny G.M. (2004). Enterococcal peptide sex pheromones: Synthesis and control of biological activity. Peptides.

[119] Clewell D.B. (1993). Bacterial sex pheromone-induced plasmid transfer. Cell.

[120] Bhardwaj A., Malik R.K., Chauan P. (2008). Functional and safety aspects of enterococci in dairy foods. Ind. J. Microbiol..

[121] Falcioni G.C., Coderoni S., Tedeschi G.G., Brunori M., Rotilio G. (1981). Red cell lysis induced by microorganisms as a case of superoxide- and hydrogen peroxide-dependent hemolysis mediated by oxyhemoglobin. Biochim. Biophys. Acta.

[122] Emerit I., Garban F., Vassy J., Levy A., Filipe P., Freitas J. (1996). Superoxide-mediated clastogenesis and anticlastogenic effects of exogenous superoxide dismutase. Proc. Natl. Acad. Sci. USA.

[123] Thompson S.L., Bakhoum S.F., Compton D.A. (2010). Mechanisms of chromosomal instability. Curr. Biol..

[124] Bakhoum S.F., Ngo B., Laughney A.M., Cavallo J.A., Murphy C.J., Ly P., Shah P., Sriram R.K., Watkins T.B.K., Taunk N.K. (2018). Chromosomal instability drives metastasis through a cytosolic DNA response. Nature.

[125] Kucerova K., Svobodova H., Tuma S., Ondrackova I., Plockova M. (2009). Production of biogenic amines by Enterococci. Czech J. Food Sci..

[126] Aspri M., Bozoudi D., Tsaltas D., Hill C., Papademas P. (2017). Raw donkey milk as a source of *Enterococcus* diversity: Assessment of their technological properties and safety characteristics. Food Control.

[127] Martın-Platero A.M., Valdivia E., Maqueda M., Martınez-Bueno M. (2009). Characterization and safety evaluation of enterococci isolated from Spanish goats’ milk cheeses. Int. J. Food Microbiol..

[128] Gimenez-Pereira M.L. (2005). Enterococci in Milk Products. Master’s Thesis.

[129] Asahara T., Takahashi M., Nomoto K., Takayama H., Onoue M., Morotomi M., Tanaka R., Yokokura T., Yamashita N. (2003). Assessment of safety of lactobacillus strains based on resistance to host innate defense mechanisms. Clin. Diagn. Lab. Immunol..

[130] Wells C.L., Jechorek R.P., Erlandsen S.L. (1990). Evidence for the translocation of *Enterococcus faecalis* across the mouse intestinal tract. J. Infect. Dis..

[131] Zou J., Shankar N. (2016). The opportunistic pathogen *Enterococcus faecalis* resists phagosome acidification and autophagy to promote intracellular survival in macrophages. Cell. Microbiol..

[132] Verneuil N., Sanguinetti M., Le Breton Y., Posteraro B., Fadda G., Auffray Y., Hartke A., Giard J.C. (2004). Effects of the *Enterococcus faecalis hyp*R gene encoding a new transcriptional regulator on oxidative stress response and intracellular survival within macrophages. Infect. Immun..

[133] Verneuil N., Rince A., Sanguinetti M., Posteraro B., Fadda G., Auffray Y., Hartke A., Giard J.C. (2005). Contribution of a PerR-like regulator to the oxidative-stress response and virulence of *Enterococcus faecalis*. Microbiology.

[134] Coburn P.S., Baghdayan A.S., Dolan G.T., Shankar N. (2008). An AraC-type transcriptional regulator encoded on the *Enterococcus faecalis* pathogenicity island contributes to pathogenesis and intracellular macrophage survival. Infect. Immun..

[135] La Carbona S., Sauvageot N., Giard J.C., Benachour A., Posteraro B., Auffray Y., Hartke A. (2007). Comparative study of the physiological roles of three peroxidases (NADH peroxidase, Alkyl hydroperoxide reductase and Thiol peroxidase) in oxidative stress response, survival inside macrophages and virulence of *Enterococcus faecalis*. Mol. Microbiol..

[136] Maekawa S., Yoshioka M., Kumamoto Y. (1992). Proposal of a new scheme for the serological typing of *Enterococcus faecalis* strains. Microbiol. Immunol..

[137] Hancock L.E., Gilmore M.S. (2002). The capsular polysaccharide of *Enterococcus faecalis* and its relationship to other polysaccharides in the cell wall. Proc. Natl. Acad. Sci. USA.

[138] Thurlow L.R., Thomas V.C., Hancock L.E. (2009). Capsular polysaccharide production in *Enterococcus faecalis* and contribution of CpsF to capsule serospecificity. J. Bacteriol..

[139] Bhardwaj A., Kapila S., Mani J., Malik R.K. (2009). Comparison of susceptibility to opsonic killing by *in vitro* human immune response of *Enterococcus* strains isolated from dairy products, clinical samples and probiotic preparation. Int. J. Food Microbiol..

[140] Liong M.T. (2008). Safety of probiotics: Translocation and infection. Nutr. Rev..

[141] Guarner C., Runyon B.A., Young S., Heck M., Seikh M.Y. (1997). Intestinal bacterial overgrowth and bacterial translocation in an experimental model of cirrhosis in rats. J. Hepatol..

[142] Chiva M., Guarner C., Peralta C., Llovet T., Gomez G., Soriano G., Balanzo J. (2003). Intestinal mucosal oxidative damage and bacterial translocation in cirrhotic rats. Eur. J. Gastroenterol. Hepatol..

[143] Manfredo Vieira S., Hiltensperger M., Kumar V., Zegarra-Ruiz D., Dehner C., Khan N., Costa F.R.C., Tiniakou E., Greiling T., Ruff W. (2018). Translocation of a gut pathobiont drives autoimmunity in mice and humans. Science.

[144] Allen T.D., Moore D.R., Wang X., Casu V., May R., Lerner M.R., Houchen C., Brackett D.J., Huycke M.M. (2008). Dichotomous metabolism of *Enterococcus faecalis* induced by haematin starvation modulates colonic gene expression. J. Med. Microbiol..

[145] Kraehenbuhl J.P., Neutra M.R. (2000). Epithelial M cells: Differentiation and function. Ann. Rev. Cell Dev. Biol..

[146] Schulz O., Jaensson E., Persson E.K., Liu X., Worbs T., Agace W.W., Pabst O. (2009). Intestinal CD103+, but not CX3CR1+, antigen sampling cells migrate in lymph and serve classical dendritic cell functions. J. Exp. Med..

[147] Knoop K.A., McDonald K.G., Kulkarni D.H., Newberry R.D. (2016). Antibiotics promote inflammation through the translocation of native commensal colonic bacteria. Gut.

[148] Stanley D., Mason L.J., Mackin K.E., Srikhanta Y.N., Lyras D., Prakash M.D., Nargali K., Venegas A., Hill M.D., Moore R.J. (2016). Translocation and dissemination of commensal bacteria in post- stroke infection. Nat. Med..

[149] Wang X., Yang Y., Huycke M.M. (2015). Commensal bacteria drive endogenous transformation and tumour stem cell marker expression through a bystander effect. Gut.

[150] Yang Y., Wang X., Moore D.R., Lightfoot S.A., Huycke M.M. (2012). TNF-α mediates macrophage-induced bystander effects through netrin-1. Cancer Res..

[151] Wang X., Allen T.D., Yang Y., Moore D.R., Huycke M.M. (2013). Cyclooxygenase-2 generates the endogenous mutagen trans-4-hydroXy-2-nonenal in *Enterococcus faecalis*-infected macrophages. Cancer Prev. Res..

[152] Balish E., Warner T. (2002). *Enterococcus faecalis* induces inflammatory bowel disease in interleukin-10 knockout mice. Am. J. Pathol..

[153] Kim S.C., Tonkonogy S.L., Albright C.A., Tsang J., Balish E.J., Braun J., Huycke M.M., Sartor R.B. (2005). Variable phenotypes of enterocolitis in IL-10 deficient mice monoassociated with two different commensal bacteria. Gastroenterology.

[154] Balamurugan R., Rajendiran E., George S., Samuel G.V., Ramakrishna B.S. (2008). Real-time polymerase chain reaction quantification of specific butyrate-producing bacteria, *Desulfovibrio* and *Enterococcus faecalis* in the feces of patients with colorectal cancer. J. Gastroenterol. Hepatol..

[155] Amarnani R., Rapose A. (2017). Colon cancer and enterococcus bacteremia coaffection: A dangerous alliance. J. Infect. Public Health.

[156] Khan Z., Siddiqui N., Saif M.W. (2018). *Enterococcus faecalis* infective endocarditis and colorectal carcinoma: Case of new association gaining ground. Gastroenterol. Res..

[157] Cabiltes I., Coghill S., Bowe S.J., Athan E. (2019). Enterococcal bacteraemia “Silent but deadly”: A population-based cohort study. Intern. Med. J..

[158] Pericàs J.M., Corredoira J., Moreno A., Garcia-Pais M.J., Falces C., Rabunal R., Mestres C.A., Alonso M.P., Marco F., Quintana E. (2017). Relationship between *Enterococcus faecalis* infective endocarditis and colorectal neoplasm: Preliminary results from a cohort of 154 patients. Rev. Esp. Cardiol. (Engl. Ed.).

[159] Lennard K.S., Goosen R.W., Blackburn J.M. (2016). Bacterially-associated transcriptional remodelling in a distinct genomic subtype of colorectal cancer provides a plausible molecular basis for disease development. PLoS ONE.

[160] O’Driscoll T., Crank C.W. (2015). Vancomycin-resistant enterococcal infections: Epidemiology, clinical manifestations, and optimal management. Infect. Drug Resist..

[161] Werner G., Coque T.M., Hammerum A.M., Hope R., Hryniewicz W., Johnson A., Klare I., Kristinsson K.G., Leclercq R., Lester C.H. (2008). Emergence and spread of vancomycin resistance among enterococci in Europe. Euro Surveill..

[162] Teixeira L.M., Merquior V.L.C. (2013). *Enterococcus*. Molecular Typing in Bacterial Infections.

[163] Mannu L., Paba A., Daga E., Comunian R., Zanetti S., Duprè I., Sechi L.A. (2003). Comparison of the incidence of virulence determinants and antibiotic resistance between *Enterococcus faecium* strains of dairy, animal and clinical origin. Int. J. Food Microbiol..

[164] Mundy L.M., Sahm D.F., Gilmore M. (2000). Relationships between enterococcal virulence and antimicrobial resistance. Clin. Microbiol. Rev..

[165] Brilliantova A.N., Kliasova G.A., Mironova A.V., Tishkov V.I., Novichkova G.A., Bobrynina V.O., Sidorenko S.V. (2010). Spread of vancomycin-resistant *Enterococcus faecium* in two haematological centres in Russia. Int. J. Antimicrob. Agents.

[166] Perin L.M., Miranda R.O., Todorov S.D., Franco B.D.G.D.M., Nero L.A. (2014). Virulence, antibiotic resistance and biogenic amines of bacteriocinogenic lactococci and enterococci isolated from goat milk. Int. J. Food Microbiol..

[167] van Harten R.M., Willems R.J.L., Martin N.I., Hendrickx A.P.A. (2017). Multidrug-resistant enterococcal infections: New compounds, novel antimicrobial therapies?. Trends Microbiol..

[168] Landete J.M., Peirotén A., Medina M., Arqués J.L., Rodríguez-Mínguez E. (2018). Virulence and antibiotic resistance of enterococci isolated from healthy breastfed infants. Microb. Drug Resist..

[169] Ch’ng J., Chong K.K., Lam L.N., Wong J.J., Kline K.A. (2019). Biofilm-associated infection by enterococci. Nat. Rev. Microbiol..

[170] Araujo T.F., Ferreira C.L.D.L.F. (2013). The genus *Enterococcus* as probiotic: Safety concerns. Braz. Arch.Biol. Technol..

[171] Kak V., Chow J.W., Gilmore M.S. (2002). Acquired antibiotic resistances in enterococci. The Enterococci: Pathogenesis, Molecular Biology, and Antibiotic Resistance.

[172] Jahan M., Holley R.A. (2016). Transfer of antibiotic resistance from *Enterococcus faecium* of fermented meat origin to Listeria monocytogenes and Listeria innocua. Lett. Appl. Microbiol..

[173] Hummel A., Holzapfel W.H., Franz C.M. (2007). Characterisation and transfer of antibiotic resistance genes from enterococci isolated from food. Syst. Appl. Microbiol..

[174] Lebreton F., Valentino M.D., Schaufler K., Earl A.M., Cattoir V., Gilmore M.S. (2018). Transferable vancomycin resistance in clade B commensal-type *Enterococcus faecium*. J. Antimicrob. Chemother..

[175] Lebreton F., Willems R.J.L., Gilmore M.S. (2014). Enterococcus diversity, origins in nature, and gut colonization. Enterococci: From Commensals to Leading Causes of Drug Resistant Infection.

[176] Franz C., Holzapfel W.H. (2004). The genus *Enterococcus*: Biotechnological and safety issues. Lactic Acid Bacteria. Microbiological and Functional Aspects.

[177] Wegener H.C. (2003). Antibiotics in animal feed and their role in resistance development. Curr. Opin. Microbiol..

[178] Leong K.W.C., Cooley L.A., Anderson T.L., Gautam S.S., McEwan B., Wells A., Wilson F., Hughson L., O’Toole R.F. (2018). Emergence of vancomycin-resistant *Enterococcus faecium* at an Australian hospital: A whole genome sequencing analysis. Sci. Rep..

[179] Guerrero-Ramos E., Molina-Gonzalez D., Blanco-Morán S., Igrejas G., Poeta P., Alonso-Calleja C., Capita R. (2016). Prevalence, antimicrobial resistance, and genotypic characterization of vancomycin-resistant enterococci in meat preparations. J. Food Prot..

[180] Aarestrup F.M., Butaye P., Witte W. (2002). Non-human reservoirs of enterococci. The Enterococci: Pathogenesis, Molecular Biology, and Antibiotic Resistance.

[181] Ronconi M.C., Merino L.A., Fernandez G. (2002). Detection of Enterococcus with high-level aminoglycoside and glycopeptide resistance in Lactuca sativa (lettuce). Enferm. Infecc. Microbiol. Clin..

[182] Bouki C., Venieri D., Diamadopoulos E. (2013). Detection and fate of antibiotic resistant bacteria in wastewater treatment plants: A review. Ecotoxicol. Environ. Saf..

[183] Huycke M.M., Gilmore M.S., Jett B.D., Booth J.L. (1992). Transfer of pheromone-inducible plasmids between *Enterococcus faecalis* in the Syrian hamster gastro-intestinal tract. J. Infect. Dis..

[184] Jacobsen B.L., Skou M., Hammerum A.M., Jensen L.B. (1999). Horizontal transfer of the satA gene encoding streptogramin A resistance between isogenic *Enterococcus faecium* strains in the gastrointestinal tract of gnotobiotic rats. Microb. Ecol. Health Dis..

[185] Lund B., Edlund C. (2001). Probiotic *Enterococcus faecium* strain is a possible recipient of the *van*A gene cluster. Clinic. Infect. Dis..

[186] Mater D.D., Langella P., Corthier G., Flores M.J. (2005). Evidence of vancomycin resistance gene transfer between enterococci of human origin in the gut of mice harbouring human microbiota. J. Antimicrob. Chemother..

[187] Coburn P.S., Baghdayan A.S., Dolan G.T., Shankar N. (2007). Horizontal transfer of virulence genes encoded on the *Enterococcus faecalis* pathogenicity island. Mol. Microbiol..

[188] Topcuoglu S., Gursoy T., Ovali F., Serce O., Karatekin G. (2015). A new risk factor for neonatal vancomycin-resistant Enterococcus colonisation: Bacterial probiotics. J. Matern. Fetal Neonatal Med..

[189] Li N., Yu H., Liu H., Wang Y., Zhou J., Ma X., Wang Z., Sun C., Qiao S. (2019). Horizontal transfer of vanA between probiotic *Enterococcus faecium* and *Enterococcus faecalis* in fermented soybean meal and in digestive tract of growing pigs. J. Anim. Sci. Biotechnol..

[190] Palmer K.L., Kos V.N., Gilmore M.S. (2010). Horizontal gene transfer and the genomics of enterococcal antibiotic resistance. Curr. Opin. Microbiol..

[191] Monticelli J., Knezevich A., Luzzati R., Di Bella S. (2018). Clinical management of non-*faecium* non-*faecalis* vancomycin-resistant enterococci infection. Focus on *Enterococcus gallinarum* and *Enterococcus casseliflavus*/*flavescens*. J. Infect. Chemother..

[192] Palmer K.L., Gilmore M.S. (2010). Multidrug-resistant enterococci lack CRISPR-cas. MBio.

[193] Hegstad K., Mikalsen T., Coque T.M., Werner G., Sundsfjord A. (2010). Mobile genetic elements and their contribution to the emergence of antimicrobial resistant Enterococcus faecalis and *Enterococcus faecium*. Clin. Microbiol. Infect..

[194] Zhang S., Lebreton F., Mansfield M.J., Miyashita S.-I., Zhang J., Schwartzman J.A., Tao L., Masuyer G., Martínez-Carranza M., Stenmark P. (2018). Identification of a Botulinum Neurotoxin-like Toxin in a Commensal Strain of *Enterococcus faecium*. Cell Host Microbe.

[195] Brunt J., Carter A.T., Stringer S.C., Peck M.W. (2018). Identification of a novel botulinum neurotoXin gene cluster in *Enterococcus*. FEBS Lett..

[196] Sharifi Y., Hasani A., Ghotaslou R., Varshochi M., Hasani A., Aghazadeh M., Milani M. (2012). Survey of virulence determinants among vancomycin resistant *Enterococcus faecalis* and *Enterococcus faecium* isolated from clinical specimens of hospitalized patients of northwest of Iran. Open Microbiol. J..

[197] Cariolato D., Andrighetto C., Lombardi A. (2008). Occurrence of virulence factors and antibiotic resistances in Enterococcus faecalis and *Enterococcus faecium* collected from dairy and human samples in North Italy. Food Control.

[198] Medeiros A.W., Pereira R.I., Oliveira D.V., Martins P.D., D’azevedo P.A., Van Der Sand S., Frazzon J., Frazzon A.P.G. (2014). Molecular detection of virulence factors among food and clinical *Enterococcus faecalis* strains in South Brazil. Braz. J. Microbiol..

[199] Ribeiro T., Oliveira M., Fraqueza M.J., Laukova A., Elias M., Tenreiro R., Barreto A.S., Semedo-Lemsaddek T. (2011). Antibiotic resistance and virulence factors among Enterococci isolated from chouriço, a traditional Portuguese dry fermented sausage. J. Food Prot..

[200] Ali S.A., Hasan K.A., Bin Asif H., Abbasi A. (2014). Environmental enterococci: I prevalence of virulence, antibiotic resistance and species distribution in poultry and its related environment in karachi Pakistan. Lett. Appl. Microbiol..

[201] Ispirli H., Demirbas F., Dertli E. (2017). Characterization of functional properties of *Enterococcus* spp. isolated from Turkish white cheese. LWT—Food Sci. Technol..

[202] Yılmaz E.S., Aslantas O., Pehlivanlar S.O., Türkyılmaz S., Kürekci C. (2016). Prevalence, antimicrobial resistance and virulence traits in enterococci from food of animal origin in Turkey. LWT—Food Sci. Technol..

[203] Abriouel H., Omar N.B., Molinos A.C., Lopez R.L., Grande M.J., Martínez-Viedma P., Ortega E., Martínez Cañamero M., Galvez A. (2008). Comparative analysis of genetic diversity and incidence of virulence factors and antibiotic resistance among enterococcal populations from raw fruit and vegetable foods, water and soil, and clinical samples. Int. J. Food Microbiol..

[204] Holmberg A., Rasmussen M. (2016). Mature biofilms of *Enterococcus faecalis* and *Enterococcus faecium* are highly resistant to antibiotics. Diagnostic Microbiol. Infect. Dis..

[205] Simoes M., Simoes L.C., Vieira M.J. (2010). A review of current and emergent biofilm control strategies. LWT—Food Sci. Technol..

[206] Chuang-Smith O.N., Wells C.L., Henry-Stanley M.J., Dunny G.M. (2010). Acceleration of *Enterococcus faecalis* biofilm formation by aggregation substance expression in an ex vivo model of cardiac valve colonization. PLoS ONE.

[207] Hufnagel M., Koch S., Creti R., Baldassarri L., Huebner J. (2004). A putative sugar-binding transcriptional regulator in a novel gene locus in Enterococcus faecalis contributes to production of biofilm and prolonged bacteremia in mice. J. Infection Dis..

[208] Mohamed J.A., Huang W., Nallapareddy S.R., Teng F., Murray B.E. (2004). Influence of origin of isolates, especially endocarditis isolates, and various genes on biofilm formation by *Enterococcus faecalis*. Infect. Immunol..

[209] Mohamed J.A., Murray B.E. (2005). Lack of correlation of gelatinase production and biofilm formation in a large collection of Enterococcus faecalis isolates. J. Clin. Microbiol..

[210] Hammad A.M., Hassan H.A., Shimamoto T. (2015). Prevalence, antibiotic resistance and virulence of *Enterococcus* spp. in Egyptian fresh raw milk cheese. Food Control.

[211] Gaspar F.B., Crespo M.T.B., Lopes M.F.S. (2009). Proposal for a reliable enterococcal cytolysin production assay avoiding apparent incongruence between phenotype and genotype. J. Med. Microbiol..

[212] Upadhyaya P.M., Ravikumar K.L., Umapathy B.L. (2009). Review of virulence factor of enterococcus: An emerging nosocomial pathogen. Ind. J. Med. Microbiol..

[213] Yoon M.Y., Kim Y.J., Hwang H.J. (2008). Properties and safety aspects of *Enterococcus faecium* strains isolated from Chungkukjang, a fermented soy product. LWT—Food Sci. Technol..

[214] Ozmen Toğay S., Celebi Keskin A., Acık L. (2010). Virulence genes, antibiotic resistance and plasmid profiles of *Enterococcus faecalis* and *Enterococcus faecium* from naturally fermented Turkish foods. J. Appl. Microbiol..

[215] Akhtar M., Hirt H., Zurek L. (2009). Horizontal transfer of the tetracycline resistance gene tetM mediated by pCF10 among *Enterococcus faecalis* in the house fly (*Musca domestica L*) alimentary canal. Microb. Ecol..

[216] Paoletti C., Foglia G., Princivalli M.S., Magi G., Guaglianone E., Donelli G., Pruzzo C., Biavasco F., Facinelli B. (2007). Co-transfer of vanA and aggregation substance genes from *Enterococcus faecalis* isolates in intra- and interspecies matings. J. Antimicrob. Chemother..

[217] Werner G., Freitas A.R., Coque T.M., Sollid J.E., Lester C., Hammerum A.M., Garcia-Migura L., Jensen L.B., Francia M.V., Witte W. (2011). Host range of enterococcal vanA plasmids among Gram-positive intestinal bacteria. J. Antimicrob. Chemother..

[218] Wardal E., Sadowy E., Hryniewicz W. (2010). Complex nature of enterococcal pheromone-responsive plasmids. Pol. J. Microbiol..

[219] Chajecka-Wierzchowska W., Zadernowska A., Łaniewska-Trokenheim Ł. (2016). Virulence factors, antimicrobial resistance and biofilm formation in *Enterococcus* spp. isolated from retail shrimps. LWT—Food Sci. Technol..

[220] Poeta P., Costa D., Saenz Y., Klibi N., Ruiz-Larrea F., Rodrigues J., Torres C. (2005). Characterization of antibiotic resistance genes and virulence factors in faecal enterococci of wild animals in Portugal. J. Vet. Med. B.

[221] Jackson C.R., Kariyawasam S., Borst L.B., Frye J.G., Barrett J.B., Hiott L.M., Woodley T.A. (2015). Antimicrobial resistance, virulence determinants and genetic profiles of clinical and nonclinical *Enterococcus cecorum* from poultry. Lett. Appl. Mirobiol..

[222] Ricci A., Allende A., Bolton D., Chemaly M., Robert D., Rosina G., Lieve H., Konstantinos K., Lindqvist R., EFSA Panel on Biological Hazards (2017). Scientific Opinion on the update of the list of QPS-recommended biological agents intentionally added to food or feed as notified to EFSA. EFSA J..

[223] Montealegre M.C., Singh K.V., Murray B.E. (2016). Gastrointestinal tract colonization dynamics by different *Enterococcus faecium* clades. J. Infect. Dis..

[224] Bonacina J., Suarez N., Hormigo R., Fadda S., Lechner M., Saavedra L. (2017). A genomic view of food-related and probiotic Enterococcus strains. DNA Res..

[225] Jung A., Metzner M., Ryll M. (2017). Comparison of pathogenic and non-pathogenic *Enterococcus cecorum* strains from different animal species. BMC Microbiol..

[226] Beukers A.G., Zaheer R., Goji N., Amoako K.K., Chaves A.V., Ward M.P., McAllister (2017). Comparative genomics of *Enterococcus* spp. isolated from bovine feces. BMC Microbiol..

[227] ACNFP (1996). Report on Enterococcus Faecium, Strain K77D Report.

[228] Lauková A., Wright A.V. (2011). Potential applications of probiotic, bacteriocin-producing enterococci and their bacteriocins. Lactic Acid Bacteria Microbiological and Functional Aspects.

[229] EFSA (2012). Guidance for assessing safety of *Enterococcus faecium* in animal feed. EFSA J..

[230] Brodmann T., Endo A., Gueimonde M., Vinderola G., Kneifel W., de Vos W.M., Salminen S., Gómez-Gallego C. (2017). Safety of novel microbes for human consumption: Practical examples of assessment in the European Union. Front. Microbiol..

[231] Nami Y., Abdullah N., Haghshenas B., Radiah D., Rosli R., Khosroushahi A.Y. (2014). Probiotic assessment of *Enterococcus durans* 6HL and Lactococcus lactis 2HL isolated from vaginal microflora. J. Med. Microbiol..

[232] Gupta A., Tiwari S.K. (2015). Probiotic potential of bacteriocin-producing *Enterococcus hirae* strain LD3 isolated from dosa batter. Ann. Microbiol..

[233] van Zyl W., Deane S., Dicks L. (2016). *Enterococcus mundtii* ST4SA and *Lactobacillus plantarum* 423 excludes *Listeria monocytogenes* from the GIT, as shown by bioluminescent studies in mice. Benef. Microbes.

[234] EFSA (2012). Scientific Opinion on Lactiferm R (*Enterococcus faecium*) as a feed additive for weaned piglets and calves. EFSA J..

[235] Laulund S., Wind A., Derkx P., Zuliani V. (2017). Regulatory and safety requirements for food cultures. Microorganisms.

[236] Hendrickx A.P., van Wamel W.J., Posthuma G., Bonten M.J., Willems R.J. (2007). Five genes encoding surface-exposed LPXTG proteins are enriched in hospital-adapted *Enterococcus faecium* clonal complex 17 isolates. J. Bacteriol..

[237] Rice L.B., Carias L., Rudin S., Vael C., Goossens H., Konstabel C., Klare I., Nallapareddy S.R., Huang W., Murray B.E. (2003). A potential virulence gene, hylEfm, predominates in *Enterococcus faecium* of clinical origin. J. Infect. Dis..

[238] Werner G., Fleige C., Geringer U., van Schaik W., Klare I., Witte W. (2011). IS element IS16 as a molecular screening tool to identify hospital-associated strains of *Enterococcus faecium*. BMC Infect. Dis..

[239] Singh K.V., Coque T.M., Weinstock G.M., Murray B.E. (1998). *In vivo* testing of an *Enterococcus faecalis* efaA mutant and use of efaA homologs for species identification. FEMS Immunol. Med. Microbiol..

[240] Teo J.W., Krishnan P., Jureen R., Lin R.T. (2011). Detection of an unusual van genotype in a vancomycin-resistant *Enterococcus faecium* hospital isolate. J. Clin. Microbiol..

[241] Nakayama J., Kariyama R., Kumon H. (2002). Description of a 23.9-kilobase chromosomal deletion containing a region encoding fsr genes which mainly determines the gelatinase-negative phenotype of clinical isolates of *Enterococcus faecalis* in urine. Appl. Environ. Microbiol..

[242] Semedo T., Almeida Santos M., Martins P., Silva Lopes M.F., Figueiredo Marques J.J., Tenreiro R., Crespo M.T.B. (2003). Comparative study using type strains and clinical and food isolates to examine hemolytic activity and occurrence of the cyl operon in enterococci. J. Clin. Microbiol..

[243] Qin X., Singh K.V., Weinstock G.M., Murray B.E. (2000). Effects of *Enterococcus faecalis fsr* genes on production of gelatinase and a serine protease and virulence. Infect. Immun..

[244] EFSA Panel on Biological Hazards (2011). Scientific opinion on risk-based control of biogenic amine formation in fermented foods. EFSA J..

[245] FDA (2011). Scombrotoxin (Histamine) Formation. Fish and Fishery Products Hazards and Controls Guidance.

[246] Hungerford J.M., Hollingworth T.A., Wekell M.M. (2001). Automated kinetics-enhanced flow-injection method for histamine in regulatory laboratories: Rapid screening and suitability requirements. Anal. Chim. Acta.

[247] Patange S.B., Mukundan M.K., Ashok Kumar K. (2005). A simple and rapid method for colorimetric determination of histamine in fish flesh. Food Control.

[248] Landete J.M., de Las Rivas B., Marcobal A., Munoz R. (2007). Molecular methods for the detection of biogenic amine-producing bacteria on foods. Int. J. Food Microbiol..

[249] Yang S.C., Lin C.H., Sung C.T., Fang J.Y. (2014). Antibacterial activities of bacteriocins: Application in foods and pharmaceuticals. Front. Microbiol..

[250] Egan K., Field D., Rea M.C., Ross R.P., Hill C., Cotter P.D. (2016). Bacteriocins: Novel solutions to age old spore-related problems?. Front. Microbiol..

[251] Vignolo G., Saavedra L., Sesma F., Raya R., Bhat R., Alias A.K., Paliyath G. (2012). Food bioprotection: Lactic acid bacteria as natural preservatives. Progress in Food Preservation.

[252] Barbosa A.A.T., Mantovani H.C., Jain S. (2017). Bacteriocins from lactic acid bacteria and their potential in the preservation of fruit products. Critic. Rev. Biotechnol..

[253] Cotter P.D., Ross R.P., Hill C. (2013). Bacteriocins-a viable alternative to antibiotics?. Nat. Rev. Microbiol..

[254] Behrens H.M., Six A., Walker D., Kleanthous C. (2017). The therapeutic potential of bacteriocins as protein antibiotics. Emerg. Topics Life Sci..

[255] Lohans C.T., Vederas J.C. (2012). Development of Class IIa bacteriocins as therapeutic agents. Int. J. Microbiol..

[256] Ołdak A., Zielinska D. (2017). Bacteriocins from lactic acid bacteria as an alternative to antibiotics. Postepy. Hig. Med. Dosw..

[257] Venugopalan V., Shriner K.A., Wong-Beringer A. (2010). Regulatory oversight and safety of probiotic use. Emerg. Infect. Dis..

[258] Hillion M., Mijouin L., Leneveu C., Jaouen T., Gérault E., Feuilloley M.G.J. Adaptation des staphylocoques commensaux aux peptides antimicrobiens de la peau. Proceedings of the 9e Congrès National de la Société Française de Microbiologie.

[259] van den Nieuwboer M., Claassen E. (2019). Dealing with the remaining controversies of probiotic safety. Benef. Microbes.

[260] Lauková A., Kandricakova A., Bunkova L., Pleva P., Scerbova J. (2017). Sensitivity to enterocins of biogenic amine-producing faecal Enterococci from ostriches and pheasants. Probiotics Antimicrob. Proteins.

[261] Grande Burgos M.J., Pulido R.P., Del Carmen Lopez Aguayo M., Galvez A., Lucas R. (2014). The cyclic antibacterial peptide enterocin AS-48: Isolation, mode of action, and possible food applications. Int. J. Mol. Sci..

[262] Jalalvand N., Esmaeili D., Bashi M.M.M., Raiszadeh M., Naeimi S. (2021). Evaluation of Physicochemical Activity of Anticancer Fusion Proteins; Enterocin A- R type pyocin-Lactocin-Ligand Against Gastric Cancer Cell Line by Real-Time RT PCR Technique. Int. J. Peptide Res. Therap..

[263] Salvucci E., Saavedra L., Hebert E., Haro C., Sesma F. (2012). Enterocin CRL35 inhibits *Listeria monocytogenes* in a murine model. Foodborne Pathog. Dis..

[264] Fritzenwanker M., Kuenne C., Billion A., Hain T., Zimmermann K., Goesmann A., Chakraborty T., Domann E. (2013). Complete genome sequence of the probiotic *Enterococcus faecalis* Symbioflor 1 Clone DSM 16431. Genome Announc..

[265] Jahan M., Zhanel G.G., Sparling R., Holley R.A. (2015). Horizontal transfer of antibiotic resistance from *Enterococcus faecium* of fermented meat origin to clinical isolates of *E. faecium* and *Enterococcus faecalis*. Int. J. Food Microbiol..

[266] de Niederhausern S., Bondi M., Messi P., Iseppi R., Sabia C., Manicardi G., Anacarso I. (2011). Vancomycin-resistance transferability from VanA enterococci to *Staphylococcus aureus*. Curr. Microbiol..

[267] Lepage E., Brinster S., Caron C., Ducroix-Crepy C., Rigottier-Gois L., Dunny G., Hennequet-Antier C., Serror P. (2006). Comparative genomic hybridization analysis of *Enterococcus faecalis*: Identification of genes absent from food strains. J. Bacteriol..

